# Accelerated biological aging and incident degenerative valvular heart disease: Findings from 408,783 UK Biobank participants

**DOI:** 10.1016/j.ijcha.2025.101838

**Published:** 2025-11-14

**Authors:** Chaoyang Lin, Enhao Wei, Qianyao Lai, Hangpan Jiang, Maosen Lin, Feng Hu, Lin Fan, Enhui Yao

**Affiliations:** a Department of Cardiology, Fujian Medical University Union Hospital, Fujian Cardiovascular Medical Center, Fujian Institute of Coronary Artery Disease, Fujian Cardiovascular Research Center, Fuzhou 350001, PR China; b School of Health, Fujian Medical University, Fuzhou, PR China; c Department of Colorectal Surgery, Clinical Oncology School of Fujian Medical University, Fuzhou, PR China

**Keywords:** PhenoAge acceleration, KDM-BA acceleration, Valvular heart disease, Aortic stenosis, Aortic regurgitation, Mitral regurgitation

## Abstract

**Background:**

Although prior studies have linked frailty and accelerated biological aging to aortic stenosis, comprehensive evidence across the spectrum of degenerative valvular diseases (VHD) and related clinical events remains unclear in middle-aged adults.

**Methods:**

We analyzed 408,783 UK Biobank participants free of baseline valvular disease. Biological age accelerations (BAA) measures were derived from clinical traits using Phenotypic Age (PhenoAge) algorithm and the Klemera-Doubal method Biological Age (KDM-BA). Outcomes included incident aortic stenosis (AS), aortic regurgitation (AR), mitral regurgitation (MR), and related interventions or mortality.

**Results:**

Over a median follow-up of 13.9 years, 10,364 incident degenerative VHD events (2.5 %) were documented, comprising 4602 AS, 1678 AS-related events, 1639 AR, and 4903 MR cases. Elevated BAA was significantly associated with higher AS risk. For PhenoAge, adjusted AS incidence rates (per 10,000 person-years; 95 % confidence interval) across quartiles (Q1–Q4) were 3.73 (3.37–4.12), 4.44 (4.05–4.88), 5.11 (4.67–5.59), and 7.79 (7.18–8.46), yielding an adjusted hazard ratio (HR) of 2.15 (1.96–2.35) for Q4. Comparable trends were observed for KDM-BA, with an adjusted HR of 1.98 (1.83–2.15) for Q4 vs Q1. AS-related events followed a similar pattern, with HRs of 1.80 (1.55–2.09) for PhenoAge Q4 and 2.22 (1.94–2.54) for KDM-BA Q4. Significant associations were also found for AR, AR-related events, and MR, but not for MR-related events.

**Conclusions:**

Among middle-aged adults, both BAA metrics were associated with increased risks of degenerative VHD and related adverse events, except for MR-related events. These findings highlight BAA as a potential tool for early risk stratification and targeted prevention.

## Highlights

• Both PhenoAge and KDM-BA acceleration are independently associated with incident AS/AR and related events.
• BAA metrics are associated with MR risk, but not with MR-related adverse events.
• BAA may serve as a novel biomarker for early risk stratification and prevention.

## Graphical abstract.

Accelerated biological aging and degenerative valvular heart disease risk. Abbreviations: VHD, valvular heart disease; AS, aortic stenosis; AR, aortic regurgitation; MR, mitral regurgitation.

**Figure:**
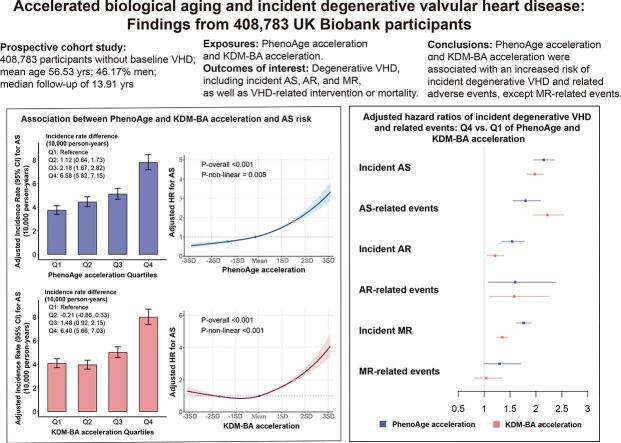

## 1 Introduction

The aging population is increasingly burdened by degenerative valvular heart disease (VHD), which has emerged as the third most prevalent cardiovascular disease globally, and aortic stenosis represents the predominant manifestation [1,2]. Despite its growing prevalence and heavy disease burden, clinical management remains challenging, as no pharmacological therapy has proven effective in halting disease progression, and surgical or transcatheter valve replacement is the only established treatment [3,4]. Early detection and intervention may reduce complications and mortality.

Chronological age is a strong risk factor for VHD. However, there exists considerable interindividual variation in the speed of valvular degeneration [1,5]. Unlike chronological age, which progresses uniformly, biological aging (BA) reflects the aggregated effects of genetic, environmental, and lifestyle elements on physiological functioning [5]. Several metrics of BA have been established, spanning from tools that utilize data from standard clinical parameters to algorithms that integrate insights throughout epigenetic, proteomic, metabolomic, and molecular analytical domains [6,7]. Among them, Klemera-Doubal method Biological Age (KDM-BA) and Phenotypic Age (PhenoAge) algorithms have demonstrated efficacy in predicting illness, functional decline, and mortality in diverse ethnic groups of older individuals [[8], [9], [10]].

Although prior studies have examined frailty or PhenoAge in relation to aortic stenosis [11,12], important gaps remain in evaluating different BA metrics and in assessing clinically relevant outcomes such as interventions and mortality. In this study, we investigated two established metrics (PhenoAge and KDM-BA) in relation to the risks of each subtype of degenerative VHD and their associated interventions and mortality in the large, prospective UK Biobank cohort. These findings offer an integrated view of the role of BA in degenerative VHD and may inform risk stratification and preventive strategies.

## 2 Method

### 2.1 Study population

**Fig. 1:**
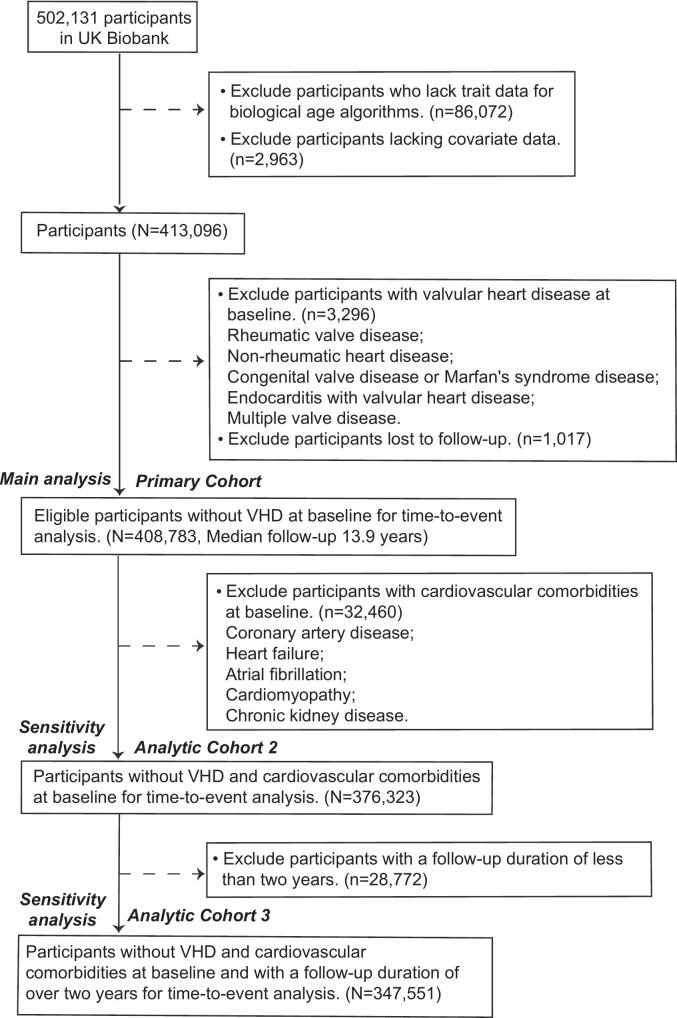
Flowchart of the study. Abbreviations: VHD, Valvular heart disease.

The UK Biobank initiative recruited participants aged 37–73 years during its baseline survey (2006–2010), collecting data from over 500,000 individuals. At baseline, participants provided information on their lifestyle, health status, and biological samples. After excluding participants with missing trait data for biological age algorithms, missing covariates, baseline valvular heart disease, or loss to follow-up, we included 408,783 participants for primary time-to-event analyses (Fig. 1). We excluded 32,460 participants with cardiovascular comorbidities (coronary artery disease, heart failure, atrial fibrillation, cardiomyopathy, or chronic kidney disease), retaining 376,323 without baseline cardiovascular comorbidities for sensitivity analysis (Analytic Cohort 2). Further sensitivity analyses excluded 28,772 participants with < 2 years follow-up, yielding a cohort of 347,551 participants (Analytic Cohort 3). This research was conducted in accordance with the Strengthening the Reporting of Observational Studies in Epidemiology (STROBE) guidelines (Supplementary Table S1).

### 2.2 Ethical review

This study adheres to the Declaration of Helsinki. Ethical approval was first provided by the NHS National Research Ethics Committee on June 17, 2011 (Reference: 11/NW/0382) and then renewed on June 18, 2021 (Reference: 21/NW/0157). All participants provided written informed consent, and none had withdrawn their consent prior to the data analysis.

### 2.3 Assessment of biological age and biological age acceleration

Biological age was calculated using two validated algorithms, the KDM-BA and PhenoAge [9,13,14], implemented via the R package BioAge with blood-chemistry-derived measures [15]. KDM-BA was computed from forced expiratory volume in one second (FEV1), systolic blood pressure, and seven blood chemistry parameters (albumin, alkaline phosphatase, blood urea nitrogen, creatinine, C-reactive protein, glycated hemoglobin, and total cholesterol). KDM-BA estimates biological age by regressing individual biomarkers against chronological age in a reference population. The algorithm integrates *n* regression lines of age modeled on *n* biomarkers, formalized as:

$$\mathrm{KDM}-BA=\frac{\sum_{i=1}^{n}{(X}_{i}-q_{i})\frac{k_{i}}{s_{i}^{2}}+\frac{\mathrm{CA}}{s_{\mathrm{BA}}^{2}}}{\sum_{i=1}^{n}{\left(\frac{k_{i}}{s_{i}}\right)}^{2}+\frac{1}{s_{\mathrm{BA}}^{2}}}$$

In this formulation, *x* corresponds to the measured value of biomarker *i* for a given participant. For each biomarker *i*, the parameters *k*, *q*, and *s* denote the regression intercept, slope, and root mean squared error, respectively. The scaling factor sBA is defined as the square root of the variance in chronological age explained by the biomarker panel within the reference population. In the BioAge package, the reference sample comprised NHANES III nonpregnant participants aged 30–75 years. We trained algorithm parameters separately for men and women.

PhenoAge was computed from nine blood chemistries including four overlapping with KDM-BA (albumin, alkaline phosphatase, creatinine, C-reactive protein, glucose, mean cell volume, red cell distribution width, white blood cell count, and lymphocyte proportion). Phenotypic age is calculated through a multivariate mortality hazard analysis using a reference population, where variables are selected via a Cox proportional hazards elastic net model with 10-fold cross-validation. The algorithm employs two parametrized Gompertz proportional-hazard models: one incorporating all 10 selected variables and another using only chronological age. The governing equation is as follows:

$$\mathrm{PhenoAge}=141.50225+\frac{\ln[-0.00553\times \ln(1-mortality\;risk)]}{0.090165}$$

Where

$$\mathrm{mortality}\;\mathrm{risk}=1-{e^{-e}}^{xb_{\left[\exp\left(120_{xy}\right)-1\right]/\gamma }};\;\gamma =0.0076927$$

$$\begin{array}{c} \mathrm{xb}=-19.907-0.0336+albumin+0.0095\times creatinine+0.1953\;\\ \;\;\;\;\;\;\;\;\;\times glucose+0.0954\times \ln\left(C-reactive\;protein\right)-0.012\;\\ \;\;\;\;\;\;\;\;\;\times lymphocyte\;percentage+0.0268\times mean\;corpuscular\;volume\;\\ \;\;\;\;\;\;\;\;\;+0.3306\times red\;cell\;distribution\;width+0.00188\times alkaline\;phosphatase\;\\ \;\;\;\;\;\;\;\;\;+0.0554\times white\;blood\;cell\;count+0.0804\times chronological\;age \end{array}$$

To quantify interindividual differences in BA, we calculated residuals from a linear regression of biological age on chronological age. These residuals, termed biological age acceleration (BAA), reflect the extent to which an individual’s physiological aging deviates from chronological age expectations (positive values indicate accelerated aging; negative values suggest decelerated aging). By design, BAA is mathematically independent of chronological age and serves as an index of biological aging. To enable comparable effect sizes between BAA measures, BAA metrics were standardized to a mean of 0 and standard deviation (SD) of 1 for continuous analyses and categorized into quartiles for dose–response assessments.

### 2.4 Assessment of valvular heart disease

The primary outcome was the incidence of common degenerative VHD, including aortic stenosis (AS), aortic regurgitation (AR), and mitral regurgitation (MR). Incident cases were identified using ICD-10 codes (AS: I35.0, I35.2; AR: I35.1; MR: I34.0) extracted from hospital records and death registries, with validation studies confirming high diagnostic accuracy for moderate-to-severe VHDs in these registries [[16], [17], [18]]. The secondary outcome comprised composite VHD-related interventions or mortality. Detailed diagnostic and procedural codes are listed in Supplementary Table S2. VHDs were defined as encompassing rheumatic, degenerative (non-rheumatic), congenital valve disease, endocarditis-associated VHD, or Marfan syndrome (Supplementary Table S3); however, the primary outcome in this study was restricted to degenerative VHD. For time-to-event analysis, individuals with any VHD (including non-degenerative subtypes) were excluded at baseline.

### 2.5 Assessment of covariates

Age was determined using cohort recruitment data. Demographic and lifestyle factors covariates included sex (men/women), ethnicity (White/non-White), education level (college degree or not), smoking status (never, former, current), alcohol intake frequency (never, month to week, daily), healthy physical activity (≥150 min/week moderate or ≥75 min/week vigorous or equivalent), and the Townsend Deprivation Index. Based on previous literature, clinical comorbidities (hypertension, obesity, dyslipidemia, diabetes, osteoporosis, coronary artery disease, heart failure, atrial fibrillation, cardiomyopathy, and chronic kidney disease) and medications (antihypertensive, lipid-lowering, anti-diabetic, and antithrombotic agents) were considered as covariates. Detailed definitions and corresponding UK Biobank variable codes are provided in Supplementary Table S3.

### 2.6 Statistical analysis

Descriptive statistics are reported as mean (standard deviation) or median [25th–75th percentile] based on the distributional skewness and kurtosis of variables. We constructed two models: Model 1 adjusted for sex, ethnicity, education, smoking status, alcohol intake frequency, physical activity, and the Townsend Deprivation Index; Model 2 additionally adjusted for clinical comorbidities (hypertension, obesity, dyslipidemia, diabetes, osteoporosis, coronary artery disease, heart failure, atrial fibrillation, cardiomyopathy, and chronic kidney disease) and medications (anti-diabetic, antithrombotic agents), excluding antihypertensive and lipid-lowering medications due to multicollinearity (variance inflation factor [VIF] > 5 for these medications when included). Clinical comorbidities and medications were considered potential mediators rather than confounders [16]; thus, their inclusion in Model 1 might obscure the true effects of BAA metrics and were reserved for secondary analysis. We calculated the crude incidence rate per 10,000 person-years, the adjusted incidence rate using estimated marginal means, and the adjusted incidence rate difference and ratio. Hazard ratios (HR) and 95 % confidence intervals (CI) were estimated using Cox proportional hazards regression. The proportional hazards assumption was validated via Schoenfeld residuals, with no violations detected. For degenerative VHD-related events, we constructed a Fine and Gray competing risks regression model, considering death from any other cause as a competing event. Dose-response relationships for incident degenerative VHDs were evaluated using restricted cubic splines regression (RCS) within Cox proportional hazards regressions, with follow-up duration as the timescale. Knots (3–7) were selected based on the minimum AIC, thereby balancing model flexibility and parsimony. Median BAA metrics served as the reference. Adjusted cumulative risk curves, generated using the *ggadjustedcurves* function (provided by *survminer* package), employed marginal analysis to visualize age-scaled survival trends. Five- and 10-year absolute risks were calculated, and follow-up time smoothing was implemented using generalized additive models (GAM) with time-parametric hazard functions; VHD-related events were modeled using generalized linear models (GLM) with other causes of death as competing events, all performed using the casebase R package.

Subgroup analyses were conducted to examine whether the associations were robust in each subgroup. Sensitivity analyses included: (1) further excluding participants with baseline cardiovascular comorbidities (coronary artery disease, heart failure, atrial fibrillation, cardiomyopathy, or chronic kidney disease) to test robustness (Analytic Cohort 2); (2) excluding those with follow-up durations < 2 years to mitigate reverse causality (Analytic Cohort 3).

All analyses were performed in R (version 4.5.0). Statistical significance was defined as two-sided *P*-value < 0.05, with Bonferroni correction (*P* < 0.0083, α = 0.05/6) for multiple testing across two BAA measures and three primary VHD outcomes (AS, AR, and MR).

## 3 Results

### 3.1 Study population and baseline characteristics

In this primary UK Biobank cohort of 408,783 participants (mean age 56.5 ± 8.1 years; 46.17 % men), 10,364 (2.54 %) developed incident degenerative VHD. Compared to non-cases, participants developing VHD were chronologically older (61.85 ± 6.20 vs. 56.39 ± 8.08 years), had a higher proportion of men (59.28 % vs. 45.83 %), and exhibited accelerated BAA [PhenoAge acceleration: 0.15 (IQR: −0.44–0.86) vs. −0.13 (IQR: −0.65–0.47); KDM-BA acceleration: 2.11 (IQR: −6.11–9.71) vs. 0.23 (IQR: −6.75–6.90)]. This group demonstrated elevated cardiovascular risk profiles (including higher rates of hypertension, diabetes, dyslipidemia, and obesity), more clinical comorbidities, and heavier medication use burden. They also demonstrated lower educational attainment and were more likely to smoke, alongside adverse metabolic profiles such as increased systolic blood pressure, elevated HbA1c, and reduced HDL-C (Table 1).

**Table1:** Characteristics of study participants.

Characteristic	Overall	Not incident degenerative VHD	Incident degenerative VHD
	N = 408,783	N = 398,419	N = 10,364
Demographics and lifestyle			
PhenoAge, years, mean (SD)	52.82 (9.35)	52.64 (9.30)	59.83 (8.52)
PhenoAge acceleration, median [IQR]	−0.12 (−0.64, 0.48)	−0.13 (−0.65, 0.47)	0.15 (−0.44, 0.86)
KDM-BA, years, mean (SD)	50.71 (13.20)	50.54 (13.13)	57.09 (14.27)
KDM-BA acceleration, median [IQR]	0.27 (−6.73, 6.97)	0.23 (−6.75, 6.90)	2.11 (−6.11, 9.71)
Chronological age, years, mean (SD)	56.53 (8.08)	56.39 (8.08)	61.85 (6.20)
Sex (men), n (%)	188,753 (46.17 %)	182,609 (45.83 %)	6144 (59.28 %)
Race (white), n (%)	371,088 (90.78 %)	361,534 (90.74 %)	9554 (92.18 %)
Education (college), n (%)	132,368 (32.38 %)	129,719 (32.56 %)	2649 (25.56 %)

Smoking status, n (%)			
Never	223,664 (54.71 %)	218,975 (54.96 %)	4689 (45.24 %)
Previous	141,862 (34.70 %)	137,365 (34.48 %)	4497 (43.39 %)
Current	43,257 (10.58 %)	42,079 (10.56 %)	1178 (11.37 %)

Alcohol intake frequency, n (%)			
Never	32,527 (7.96 %)	31,531 (7.91 %)	996 (9.61 %)
Month to week	292,722 (71.61 %)	285,656 (71.70 %)	7066 (68.18 %)
Daily	83,534 (20.43 %)	81,232 (20.39 %)	2302 (22.21 %)
Healthy physical activity (yes), n (%)	221,338 (54.15 %)	215,990 (54.21 %)	5348 (51.60 %)
Townsend deprivation index, median [IQR]	−2.16 (−3.66, 0.49)	−2.16 (−3.66, 0.49)	−2.06 (−3.59, 0.73)

Comorbidities			
Hypertension, n (%)	139,858 (34.21 %)	133,772 (33.58 %)	6086 (58.72 %)
Obesity, n (%)	10,751 (2.63 %)	10,263 (2.58 %)	488 (4.71 %)
Dyslipidaemia, n (%)	118,111 (28.89 %)	112,532 (28.24 %)	5579 (53.83 %)
Diabetes, n (%)	25,572 (6.26 %)	24,111 (6.05 %)	1461 (14.10 %)
Osteoporosis, n (%)	8,492 (2.08 %)	8,172 (2.05 %)	320 (3.09 %)
Coronary artery disease, n (%)	21,328 (5.22 %)	19,646 (4.93 %)	1682 (16.23 %)
Heart failure, n (%)	1,749 (0.43 %)	1,521 (0.38 %)	228 (2.20 %)
Atrial fibrillation, n (%)	6,069 (1.48 %)	5,427 (1.36 %)	642 (6.19 %)
Cardiomyopathy, n (%)	3,937 (0.96 %)	3,699 (0.93 %)	238 (2.30 %)
Chronic kidney disease, n (%)	4,657 (1.14 %)	4,353 (1.09 %)	304 (2.93 %)

Medications			
Antihypertensive medication, n (%)	107,826 (26.38 %)	102,624 (25.76 %)	5202 (50.19 %)
Lipid-lowering medication, n (%)	109,894 (26.88 %)	104,537 (26.24 %)	5357 (51.69 %)
Anti-diabetic medication, n (%)	15,018 (3.67 %)	14,054 (3.53 %)	964 (9.30 %)
Antithrombotic medication, n (%)	61,083 (14.94 %)	57,484 (14.43 %)	3599 (34.73 %)

Physical measures and biomarkers			
Systolic blood pressure, mmHg, mean (SD)	137.94 (18.60)	137.76 (18.54)	144.68 (19.59)
Diastolic blood pressure, mmHg, mean (SD)	82.33 (10.12)	82.33 (10.11)	82.38 (10.64)
Body mass index, kg/m^2^, median [IQR]	26.70 (24.20, 29.90)	26.70 (24.10, 29.80)	28.00 (25.30, 31.50)
LDL direct, mmol/L, mean (SD)	3.56 (0.87)	3.57 (0.87)	3.43 (0.94)
HDL, mmol/L, mean (SD)	1.45 (0.38)	1.45 (0.38)	1.37 (0.37)
Lipoprotein(a), nmol/L, median [IQR]	21.10 (9.58, 61.93)	21.01 (9.56, 61.70)	24.10 (10.04, 74.60)
Triglycerides, mmol/L, median [IQR]	1.48 (1.05, 2.15)	1.48 (1.04, 2.14)	1.63 (1.16, 2.32)
HbA1c, %, mean (SD)	5.45 (0.61)	5.45 (0.60)	5.67 (0.85)
eGFR, mL/min/1.73 m^2^, median [IQR]	97.28 (87.19, 103.76)	97.36 (87.33, 103.87)	94.12 (81.90, 99.65)

Categorical variables are presented as numbers (percentages), and continuous variables as means (standard deviations) or medians (interquartile ranges), as appropriate. Abbreviations: SD, standard deviation; IQR, interquartile range; VHD, valvular heart disease; LDL, low-density lipoprotein; HDL, high-density lipoprotein; HbA1c, glycated hemoglobin; eGFR, estimated glomerular filtration rate.

### 3.2 Biological aging and risk of aortic valve stenosis and related events

**Fig. 2:**
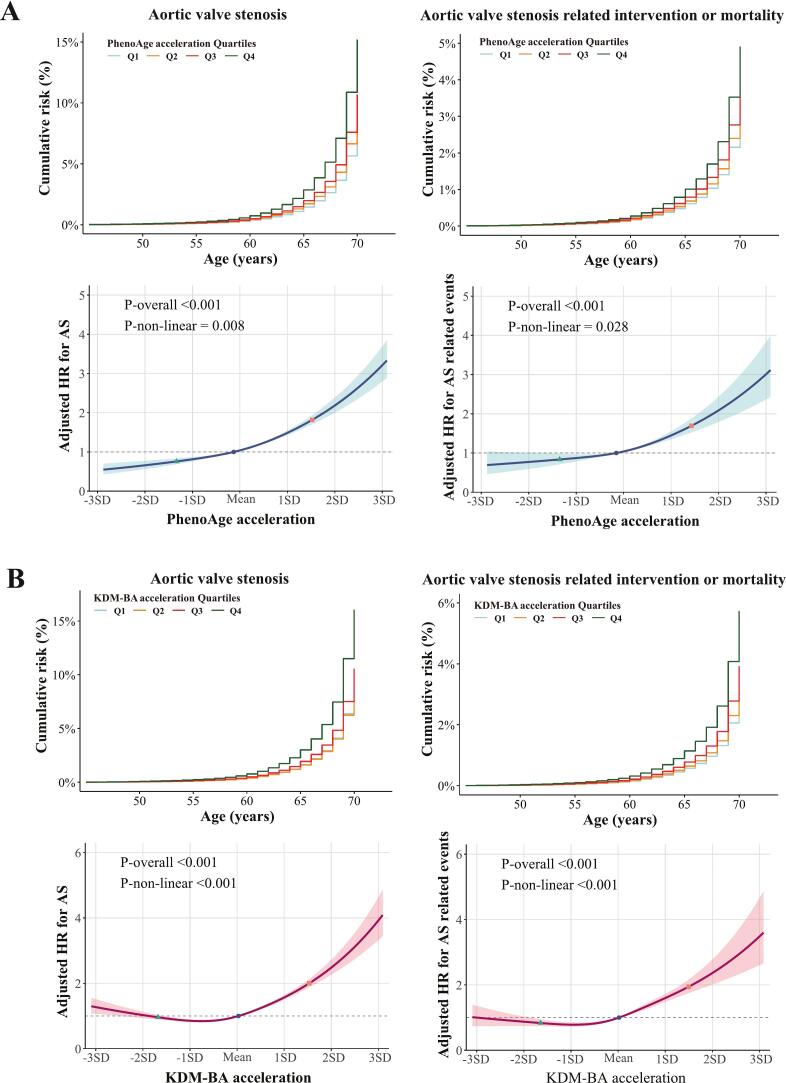
Adjusted survival curves and dose–response relationships between BAA and the risk of AS and related events. (A) Associations of PhenoAge acceleration with AS and AS-related events (intervention or mortality). (B) Associations of KDM-BA acceleration with AS and AS-related events. Models were adjusted for age, sex, ethnicity, education, smoking status, alcohol intake frequency, physical activity, and Townsend deprivation index. Upper panel: The adjusted cumulative risk plot was generated using age as the timescale. Lower panel: The restricted cubic spline plot was generated using follow-up duration as the timescale. Solid line: Point estimate; Shaded area: 95% confidence interval; Dots: 5th, 50th, and 95th percentiles. Abbreviations: AS, aortic stenosis; BAA, biological age acceleration; PhenoAge, phenotypic age; KDM-BA, Klemera–Doubal method biological age; HR, hazard ratio; SD, standard deviation.

BAA metrics showed non-linear associations with the risk of AS and AS-related intervention or mortality (All *P*-non-linear < 0.001; Fig. 2). Over a median follow-up of 13.9 years, 4,602 participants developed incident AS and 1678 experienced AS-related intervention or death, yielding crude incidence rates of 8.40 (95 % CI: 8.16–8.65) and 3.24 (95 % CI: 3.09–3.40) per 10,000 person-years, respectively (Table 2). For PhenoAge acceleration, the adjusted incidence rates of AS across quartiles (Q1 to Q4) were 3.73 (95 % CI: 3.37–4.12), 4.44 (95 % CI: 4.05–4.88), 5.11 (95 % CI: 4.67–5.59), and 7.79 (95 % CI: 7.18–8.46) per 10,000 person-years. Compared with Q1, the adjusted HRs for AS were 1.20 (95 % CI: 1.08–1.32) for Q2, 1.38 (95 % CI: 1.26–1.52) for Q3, and 2.15 (95 % CI: 1.96–2.35) for Q4. For AS-related events, the adjusted incidence rates across PhenoAge acceleration quartiles were 1.55 (95 % CI: 1.32–1.82), 1.70 (95 % CI: 1.46–1.99), 1.99 (95 % CI: 1.72–2.31), and 2.81 (95 % CI: 2.44–3.23) per 10,000 person-years, with corresponding adjusted HRs of 1.10 (95 % CI: 0.94–1.28) for Q2, 1.28 (95 % CI: 1.10–1.49) for Q3, and 1.80 (95 % CI: 1.55–2.09) for Q4, respectively.

**Table 2:** Incidence metrics and hazard ratios for aortic valve stenosis and related events across BAA quartiles.

		PhenoAge acceleration Quartiles				
		Total	Q1	Q2	Q3	Q4
Aortic valve stenosis	Event, n (%)	4602 (1.13 %)	710 (0.69 %)	904 (0.88 %)	1120 (1.10 %)	1868 (1.84 %)
	Incidence rate ^a^	8.40 (8.16, 8.65)	5.08 (4.72, 5.47)	6.52 (6.11, 6.96)	8.15 (7.69, 8.65)	14.17 (13.54, 14.83)
	Adjusted incidence rate ^a,b^	−	3.73 (3.37, 4.12)	4.44 (4.05, 4.88)	5.11 (4.67, 5.59)	7.79 (7.18, 8.46)
	Adjusted incidence rate difference ^a,b^	−	ref	**1.12 (0.64, 1.73)**	**2.18 (1.67, 2.82)**	**6.58 (5.82, 7.15)**
	Adjusted incidence rate ratio ^b^	−	ref	**1.20 (1.11, 1.31)**	**1.38 (1.28, 1.52)**	**2.15 (1.98, 2.32)**
	Adjusted hazard ratio ^b^	−	ref	**1.20 (1.08, 1.32)**	**1.38 (1.26, 1.52)**	**2.15 (1.96, 2.35)**

Aortic valve stenosis-related intervention or mortality	Event, n (%)	1678 (0.41 %)	284 (0.28 %)	345 (0.34 %)	434 (0.42 %)	615 (0.60 %)
	Incidence rate ^a^	3.24 (3.09, 3.40)	2.11 (1.87, 2.36)	2.59 (2.33, 2.88)	3.32 (3.03, 3.65)	5.14 (4.75, 5.57)
	Adjusted incidence rate ^a,b^	−	1.55 (1.32, 1.82)	1.70 (1.46, 1.99)	1.99 (1.72, 2.31)	2.81 (2.44, 3.23)
	Adjusted incidence rate difference ^a,b^	−	ref	0.25 (−0.25, 0.63)	**0.71 (0.3, 1.13)**	**2.02 (1.49, 2.45)**
	Adjusted incidence rate ratio ^b^	−	ref	1.10 (0.90, 1.28)	**1.29 (1.11, 1.50)**	**1.82 (1.56, 2.14)**
	Adjusted hazard ratio ^b^	−	ref	1.10 (0.94, 1.28)	**1.28 (1.10, 1.49)**	**1.80 (1.55, 2.09)**

		KDM-BA acceleration Quartiles				
		Total	Q1	Q2	Q3	Q4
Aortic valve stenosis	Event, n (%)	4602 (1.13%)	995 (0.98%)	817 (0.80%)	1048 (1.02%)	1742 (1.71%)
	Incidence rate ^a^	8.40 (8.16, 8.65)	7.28 (6.84, 7.74)	5.91 (5.51, 6.32)	7.61 (7.16, 8.09)	12.91 (12.32, 13.53)
	Adjusted incidence rate ^a,b^	−	4.07 (3.71, 4.47)	3.95 (3.59, 4.35)	5.00 (4.57, 5.48)	7.99 (7.36, 8.67)
	Adjusted incidence rate difference ^a,b^	−	ref	−0.21 (−0.86, 0.33)	**1.48 (0.92, 2.15)**	**6.40 (5.66, 7.03)**
	Adjusted incidence rate ratio ^b^	−	ref	0.97 (0.88, 1.05)	**1.23 (1.13, 1.35)**	**1.99 (1.86, 2.14)**
	Adjusted hazard ratio ^b^	−	ref	0.97 (0.88, 1.06)	**1.23 (1.12, 1.34)**	**1.98 (1.83, 2.15)**
Aortic valve stenosis-related intervention or mortality	Event, n (%)	1678 (0.41%)	351 (0.34%)	317 (0.31%)	401 (0.39%)	609 (0.60%)
	Incidence rate ^a^	3.24 (3.09, 3.40)	2.71 (2.44, 3.01)	2.39 (2.14, 2.67)	3.06 (2.77, 3.37)	4.86 (4.49, 5.26)
	Adjusted incidence rate ^a,b^	−	1.40 (1.19, 1.64)	1.51 (1.29, 1.77)	1.97 (1.70, 2.29)	3.10 (2.70, 3.55)
	Adjusted incidence rate difference ^a,b^	−	ref	0.19 (−0.18, 0.57)	**0.95 (0.58, 1.36)**	**2.82 (2.25, 3.24)**
	Adjusted incidence rate ratio ^b^	−	ref	1.08 (0.93, 1.26)	**1.41 (1.25, 1.64)**	**2.22 (1.92, 2.46)**
	Adjusted hazard ratio ^b^	−	ref	1.08 (0.93, 1.26)	**1.41 (1.22, 1.63)**	**2.22 (1.94, 2.54)**

^a^Per 10,000 person-years (95 % confidence interval).

^b^Adjusted for age, sex, ethnicity, education, smoking status, alcohol intake frequency, healthy physical activity, and Townsend deprivation index.

Adjusted incidence rate difference, adjusted incidence rate ratio, and adjusted hazard ratio in bold represent statistical significance (*P* < 0.05).

Abbreviations: BAA, biological age acceleration; PhenoAge, phenotypic age; KDM-BA, Klemera–Doubal method biological age.

Consistent trends were observed for KDM-BA acceleration. The adjusted incidence rates of AS across KDM-BA acceleration quartiles were 4.07 (95 % CI: 3.71–4.47), 3.95 (95 % CI: 3.59–4.35), 5.00 (95 % CI: 4.57–5.48), and 7.99 (95 % CI: 7.36–8.67) per 10,000 person-years, with adjusted HRs of 0.97 (95 % CI: 0.88–1.06) for Q2, 1.23 (95 % CI: 1.12–1.34) for Q3, and 1.98 (95 % CI: 1.83–2.15) for Q4. For AS-related events, the adjusted incidence rates across KDM-BA acceleration quartiles were 1.40 (95 % CI: 1.19–1.64), 1.51 (95 % CI: 1.29–1.77), 1.97 (95 % CI: 1.70–2.29), and 3.10 (95 % CI: 2.70–3.55) per 10,000 person-years, with adjusted HRs of 1.08 (95 % CI: 0.93–1.26) for Q2, 1.41 (95 % CI: 1.22–1.63) for Q3, and 2.22 (95 % CI: 1.94–2.54) for Q4, respectively.

Higher BAA was associated with elevated absolute 5-year and 10-year risks of AS and AS-related events among both male and female participants (Table 4). Among men and women aged 60–70 years, the 10-year AS risks were 2.92 % and 1.75 % for PhenoAge acceleration > 75th percentile, 1.76 % and 1.03 % for the 25th–75th percentile, and 1.35 % and 0.79 % for the < 25th percentile. Corresponding 10-year risks of AS-related events were 0.88 % and 0.42 %, 0.68 % and 0.32 %, and 0.58 % and 0.27 %. Similar trends were observed across KDM-BA acceleration groups.

### 3.3 Biological aging and risk of aortic regurgitation, mitral regurgitation and related events

**Fig. 3:**
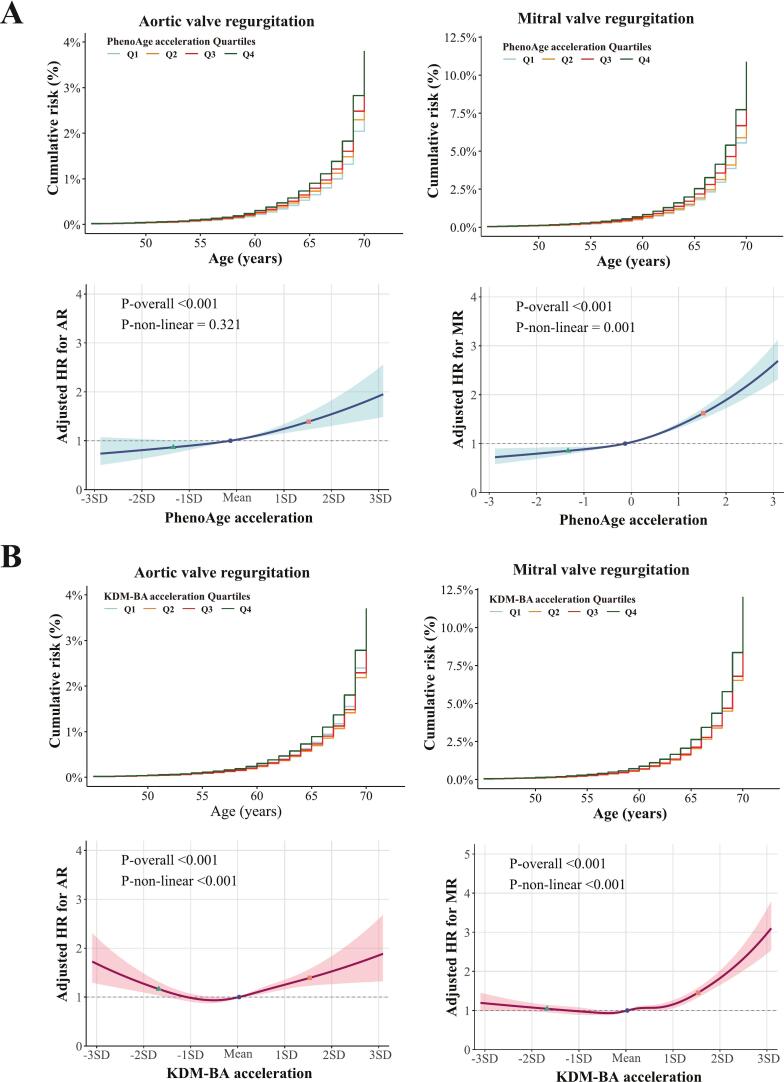
Adjusted survival curves and dose–response relationships between BAA and the risk of AR and MR. (A) Associations of PhenoAge acceleration with AR and MR. (B) Associations of KDM-BA acceleration with AR and MR. Models were adjusted for age, sex, ethnicity, education, smoking status, alcohol intake frequency, physical activity, and Townsend deprivation index. Upper panel: The adjusted cumulative risk plot was generated using age as the timescale. Lower panel: The restricted cubic spline plot was generated using follow-up duration as the timescale. Solid line: Point estimate; shaded area: 95 % confidence interval; dots: 5th, 50th, and 95th percentiles. Abbreviations: AR, aortic regurgitation; MR, mitral regurgitation; BAA, biological age acceleration; PhenoAge, phenotypic age; KDM-BA, Klemera–Doubal method biological age; HR, hazard ratio; SD, standard deviation.

The non-linear associations between BAA metrics and the risk of AR and MR were significant except for that between PhenoAge acceleration and AR (*P*-non-linear = 0.321), with 1,639 and 4,903 incident events observed respectively (Fig. 3 and Table 3). For PhenoAge acceleration, adjusted AR incidence rates rose from 2.28 (95 % CI: 1.97–2.63) for Q1 to 3.41 (95 % CI: 3.01–3.87) for Q4 per 10,000 person-years, with an adjusted HR of 1.54 (95 % CI: 1.33–1.78) for Q4. Adjusted MR incidence rates rose from 5.98 (95 % CI: 5.48–6.53) for Q1 to 10.25 (95 % CI: 9.51–11.04) for Q4 per 10,000 person-years, yielding an adjusted HR of 1.76 (95 % CI: 1.62–1.91) for Q4. KDM-BA acceleration exhibited comparable trends. Among men and women aged 60–70 years, the 10-year risks for AR were 0.49 % and 0.34 % for PhenoAge acceleration > 75th percentile, 0.38 % and 0.26 % for the 25th–75th percentile, and 0.32 % and 0.22 % for the < 25th percentile. Corresponding 10-year risks of MR were 1.72 % and 1.31 %, 1.12 % and 0.84 %, and 0.98 % and 0.73 %. Similar trends were observed in the KDM-BA acceleration groups.

**Table 3:** Incidence metrics and hazard ratios for aortic valve regurgitation and mitral valve regurgitation across BAA quartiles.

		PhenoAge acceleration Quartiles				
		Total	Q1	Q2	Q3	Q4
Aortic valve regurgitation	Event, n (%)	1639 (0.40 %)	320 (0.31 %)	368 (0.36 %)	421 (0.41 %)	530 (0.52 %)
	Incidence rate ^a^	2.99 (2.84, 3.13)	2.29 (2.05, 2.55)	2.65 (2.39, 2.93)	3.06 (2.78, 3.36)	4.00 (3.68, 4.36)
	Adjusted incidence rate ^a,b^	−	2.28 (1.97, 2.63)	2.54 (2.21, 2.92)	2.80 (2.45, 3.20)	3.41 (3.01, 3.87)
	Adjusted incidence rate difference ^a,b^	−	ref	0.29 (−0.14, 0.64)	**0.57 (0.22, 0.88)**	**1.29 (0.83, 1.73)**
	Adjusted incidence rate ratio ^b^	−	ref	1.12 (0.95, 1.29)	**1.24 (1.09, 1.40)**	**1.54 (1.32, 1.77)**
	Adjusted hazard ratio ^b^	−	ref	1.12 (0.96, 1.30)	**1.24 (1.07, 1.44)**	**1.54 (1.33, 1.78)**

Mitral valve regurgitation	Event, n (%)	4903 (1.20 %)	945 (0.92 %)	1042 (1.02 %)	1167 (1.14 %)	1749 (1.72 %)
	Incidence rate ^a^	8.95 (8.71, 9.21)	6.77 (6.35, 7.21)	7.52 (7.07, 7.99)	8.50 (8.02, 9.00)	13.26 (12.65, 13.90)
	Adjusted incidence rate ^a,b^	−	5.98 (5.48, 6.53)	6.51 (5.98, 7.08)	7.06 (6.51, 7.67)	10.25 (9.51, 11.04)
	Adjusted incidence rate difference ^a,b^	−	ref	0.65 (−0.11, 1.28)	**1.33 (0.64, 1.85)**	**5.30 (4.53, 6.04)**
	Adjusted incidence rate ratio ^b^	−	ref	1.09 (0.98, 1.19)	**1.19 (1.08, 1.27)**	**1.76 (1.61, 1.90)**
	Adjusted hazard ratio ^b^	−	ref	**1.09 (1.0003, 1.19)**	**1.19 (1.09, 1.30)**	**1.76 (1.62, 1.91)**

		KDM-BA acceleration Quartiles				
		Total	Q1	Q2	Q3	Q4
Aortic valve regurgitation	Event, n (%)	1639 (0.40%)	430 (0.42%)	342 (0.33%)	384 (0.38%)	483 (0.47%)
	Incidence rate ^a^	2.99 (2.84, 3.13)	3.14 (2.86, 3.45)	2.47 (2.22, 2.74)	2.78 (2.52, 3.08)	3.57 (3.26, 3.90)
	Adjusted incidence rate ^a,b^	−	2.72 (2.38, 3.11)	2.41 (2.1, 2.78)	2.67 (2.33, 3.06)	3.26 (2.87, 3.70)
	Adjusted incidence rate difference ^a,b^	−	ref	−0.33 (−0.71, 0.06)	−0.05 (−0.50, 0.28)	**0.60 (0.17, 1.00)**
	Adjusted incidence rate ratio ^b^	−	ref	0.89 (0.78, 1.02)	0.98 (0.85, 1.10)	**1.21 (1.05, 1.36)**
	Adjusted hazard ratio ^b^	−	ref	0.89 (0.77, 1.02)	0.98 (0.85, 1.13)	**1.21 (1.06, 1.38)**
Mitral valve regurgitation	Event, n (%)	4903 (1.20%)	1195 (1.17%)	1041 (1.02%)	1151 (1.13%)	1516 (1.48%)
	Incidence rate ^a^	8.95 (8.71, 9.21)	8.75 (8.26, 9.26)	7.53 (7.09, 8.00)	8.36 (7.89, 8.86)	11.23 (10.68, 11.81)
	Adjusted incidence rate ^a,b^	−	6.90 (6.35, 7.49)	6.67 (6.13, 7.26)	7.23 (6.66, 7.85)	9.24 (8.56, 9.97)
	Adjusted incidence rate difference ^a,b^	−	ref	−0.28 (−0.87, 0.27)	0.39 (−0.21, 1.07)	**2.85 (2.29, 3.42)**
	Adjusted incidence rate ratio ^b^	−	ref	0.97 (0.90, 1.03)	1.05 (0.98, 1.14)	**1.35 (1.28, 1.44)**
	Adjusted hazard ratio ^b^	−	ref	0.97 (0.89, 1.05)	1.05 (0.97, 1.14)	**1.35 (1.25, 1.46)**

^a^Per 10 000 person-years (95 % confidence interval).

^b^Adjusted for age, sex, ethnicity, education, smoking status, alcohol intake frequency, healthy physical activity, and Townsend deprivation index.

Adjusted incidence rate difference, adjusted incidence rate ratio, and adjusted hazard ratio in bold represent statistical significance (*P* < 0.05).

Abbreviations: BAA, biological age acceleration; PhenoAge, phenotypic age; KDM-BA, Klemera–Doubal method biological age.

**Table 4:** The 5-year and 10-year absolute risks of degenerative VHD across BAA quartiles.

		Aortic valve stenosis			Aortic valve stenosis related events		
		Women	Men	Age	Women	Men	Age
PhenoAge acceleration	<25th	0.03 % → 0.07 %	0.06 % → 0.12 %		0.02 % → 0.03 %	0.04 % → 0.06 %	
	25th-75th	0.05 % → 0.09 %	0.08 % → 0.16 %	40–50	0.02 % → 0.04 %	0.04 % → 0.08 %	40–50
	>75th	0.08 % → 0.16 %	0.14 % → 0.27 %		0.03 % → 0.05 %	0.06 % → 0.10 %	
	<25th	0.13 % → 0.25 %	0.22 % → 0.44 %		0.07 % → 0.12 %	0.14 % → 0.24 %	
	25th-75th	0.17 % → 0.33 %	0.29 % → 0.58 %	50–60	0.08 % → 0.14 %	0.16 % → 0.29 %	50–60
	>75th	0.30 % → 0.58 %	0.51 % → 0.99 %		0.10 % → 0.18 %	0.21 % → 0.37 %	
	<25th	0.40 % → 0.79 %	0.69 % → 1.35 %		0.15 % → 0.27 %	0.33 % → 0.58 %	
	25th-75th	0.53 % → 1.03 %	0.91 % → 1.76 %	60–70	0.18 % → 0.32 %	0.38 % → 0.68 %	60–70
	>75th	0.91 % → 1.75 %	1.55 % → 2.92 %		0.23 % → 0.42 %	0.50 % → 0.88 %	

KDM-BA acceleration							
	<25th	0.03 % → 0.07 %	0.08 % → 0.15 %		0.01 % → 0.02 %	0.03 % → 0.06 %	
	25th-75th	0.04 % → 0.08 %	0.08 % → 0.17 %	40–50	0.02 % → 0.03 %	0.04 % → 0.08 %	40–50
	>75th	0.07 % → 0.14 %	0.16 % → 0.31 %		0.03 % → 0.05 %	0.07 % → 0.12 %	
	<25th	0.13 % → 0.25 %	0.27 % → 0.54 %		0.05 % → 0.09 %	0.13 % → 0.23 %	
	25th-75th	0.14 % → 0.28 %	0.30 % → 0.60 %	50–60	0.07 % → 0.12 %	0.16 % → 0.29 %	50–60
	>75th	0.26 % → 0.52 %	0.57 % → 1.11 %		0.11 % → 0.19 %	0.26 % → 0.46 %	
	<25th	0.40 % → 0.78 %	0.86 % → 1.66 %		0.13 % → 0.22 %	0.31 % → 0.54 %	
	25th-75th	0.44 % → 0.87 %	0.96 % → 1.84 %	60–70	0.16 % → 0.28 %	0.39 % → 0.69 %	60–70
	>75th	0.82 % → 1.60 %	1.76 % → 3.32 %		0.25 % → 0.45 %	0.62 % → 1.09 %	

		Aortic valve regurgitation			Mitral valve regurgitation		
		Women	Men	Age	Women	Men	Age
PhenoAge acceleration	<25th	0.01% → 0.04%	0.02% → 0.06%	40–50	0.04% → 0.13%	0.06% → 0.17%	40–50
	25th-75th	0.02% → 0.05%	0.02% → 0.08%		0.05% → 0.15%	0.07% → 0.20%	
	>75th	0.02% → 0.07%	0.03% → 0.10%		0.08% → 0.24%	0.11% → 0.32%	
						
	<25th	0.03% → 0.11%	0.05% → 0.16%	50–60	0.11% → 0.34%	0.15% → 0.45%	50–60
	25th-75th	0.04% → 0.13%	0.06% → 0.19%		0.13% → 0.39%	0.18% → 0.52%	
	>75th	0.05% → 0.17%	0.08% → 0.25%		0.21% → 0.61%	0.28% → 0.82%	
						
	<25th	0.07% → 0.22%	0.10% → 0.32%	60–70	0.25% → 0.73%	0.34% → 0.98%	60–70
	25th-75th	0.08% → 0.26%	0.12% → 0.38%		0.29% → 0.84%	0.39% → 1.12%	
	>75th	0.11% → 0.34%	0.16% → 0.49%		0.46% → 1.31%	0.62% → 1.72%	
							
KDM-BAacceleration							
	<25th	0.02% → 0.05%	0.03% → 0.08%	40–50	0.05% → 0.14%	0.08% → 0.22%	40–50
	25th-75th	0.01% → 0.05%	0.02% → 0.08%		0.05% → 0.14%	0.08% → 0.22%	
	>75th	0.02% → 0.06%	0.03% → 0.10%		0.07% → 0.20%	0.11% → 0.31%	
						
	<25th	0.04% → 0.12%	0.06% → 0.20%	50–60	0.12% → 0.36%	0.19% → 0.56%	50–60
	25th-75th	0.04% → 0.11%	0.06% → 0.19%		0.12% → 0.36%	0.19% → 0.56%	
	>75th	0.05% → 0.15%	0.08% → 0.25%		0.17% → 0.50%	0.27% → 0.77%	
						
	<25th	0.08% → 0.25%	0.13% → 0.40%	60–70	0.27% → 0.79%	0.42% → 1.20%	60–70
	25th-75th	0.08% → 0.23%	0.12% → 0.38%		0.28% → 0.80%	0.43% → 1.22%	
	>75th	0.10% → 0.31%	0.16% → 0.49%		0.38% → 1.09%	0.59% → 1.64%	

The left side of the arrow indicates the 5-year absolute risk, while the right side indicates the 10-year absolute risk.

Abbreviations: BAA, biological age acceleration; PhenoAge, phenotypic age; KDM-BA, Klemera–Doubal method biological age; VHD, valvular heart disease.

For AR-related intervention or mortality, event numbers were sparse (n = 244) but showed significant associations in higher quartiles of two BAAs (PhenoAge and KDM-BA), with no non-linear associations observed (Supplementary Fig. S1 and Table S4). For instance, adjusted HRs for AR-related events in the highest quartile (Q4) were 1.60 (95 % CI: 1.08–2.39) for PhenoAge acceleration and 1.58 (95 % CI: 1.10–2.26) for KDM-BA acceleration. Furthermore, BAA metrics and MR-related events (n = 510) exhibited no significant associations.

### 3.4 Additional analysis

The associations between two BAA metrics and each valvular phenotype (AS, AS-related events, AR, and MR) risk remained robust across age, sex, education, lifestyle, and comorbidity strata (Supplementary Table S5). Specifically, while men exhibited a higher absolute risk for VHD outcomes, the relative associations between BAA and increased VHD risk were consistent in both sexes (Table 4). Sensitivity analyses confirmed the robustness of the results. After full adjustment for clinical comorbidities and medications (Model 2), adjusted HRs for AS, AS-related events, AR, MR, and their related events increased monotonically across quartiles (Supplementary Fig. S2). Furthermore, analyses excluding participants with baseline cardiovascular comorbidities (Supplementary Fig. S3) and with follow-up duration < 2 years (Supplementary Fig. S4) yielded robust results. Multicollinearity diagnostics (Supplementary Table S6) showed VIF < 2.75 for all covariates, indicating no severe collinearity.

## 4 Discussion

In this large prospective cohort study, we found that higher BAA metrics (measured by PhenoAge and KDM-BA algorithms) were associated with increased risks of degenerative VHD, including AS, AR, and MR, and were also associated with AS- and AR-related clinical events but not with MR-related events. These associations remained robust across high-risk subgroups such as older adults and individuals with hypertension, diabetes, or obesity. We also identified non-linear relationships between BAA metrics and the risks of AS, AR, and MR, as well as with AS-related events. Collectively, our findings provide novel insights into the role of BAA in the development of degenerative VHD, underscoring its potential utility for early detection and risk stratification. While chronological age remains a dominant risk factor, BAA offers additional prognostic information and helps explain interindividual differences in disease susceptibility among individuals of the same chronological age.

BAA metrics quantify the discrepancy between biological and chronological age and more accurately reflect systemic physiological decline than simple chronology. They have also emerged as strong predictive indicators for cardiovascular events [19,20]. In this study, we employed both the KDM-BA and PhenoAge algorithms, which have been validated as reliable measures of BA that capture multisystem senescence [6,21]. Prior UK Biobank studies have reported that heightened BAA (PhenoAge and KDM-BA accelerations) correlate with decreased left ventricular mass, compromised global longitudinal strain, and reduced ventricular and atrial stroke volumes at baseline [22]. BAA metrics (not limited to the aforementioned algorithms) were positively associated with multiple cardiovascular diseases, such as incident T2D [23], CAD [23], heart failure [22], stroke [24], and atrial fibrillation [25]. Our findings reveal that higher BAA metrics are intrinsically associated with an increased incidence of degenerative VHDs, and this association remains robust across different subgroups. This indicates that BAA metrics exhibit promising predictive value for degenerative VHDs risk, particularly in identifying high-risk individuals with premature aging phenotypes. A bidirectional Mendelian randomisation study revealed that epigenetic age acceleration derived from HorvathAge and PhenoAge causally elevates the risk of AS in individuals of European ancestry, further validating the causative role of BAA metrics in the development of AS from a genetic perspective [11].

Although individual biomarkers provide insights, the integration of multiple biomarkers may offer a holistic understanding of the complexity of aging. These two algorithms differ in the parameters included but both encompass markers of inflammation (C-reactive protein) and kidney function (creatinine). BAA metrics correlate with systemic inflammatory burden. Previous studies have demonstrated that local mechanical and biochemical stimuli induce chronic inflammation, which in turn activates valvular interstitial cells (VICs) and promotes valvular fibrosis and calcification [26]. Lipid-driven inflammation and VIC senescence contribute to fibrocalcific remodeling in AS [27]. While CKD and AS share overlapping risk factors, CKD is independently linked to AS progression, with an inverse association between declining estimated glomerular filtration rate (eGFR) and incident AS [28]. Studies show early-stage CKD-associated endothelial dysfunction causes plasma accumulation of inflammatory cells and molecules (including harmful cholesterol such as oxidized LDL and lipoprotein(a), minerals [calcium, phosphorus], and bone metabolism factors) that infiltrate the aortic valve, inducing thickening and calcification [29]. Moreover, CKD rapidly accelerates AS progression: dialysis patients exhibit an annual decline in aortic valve area nearly triple that of non-CKD individuals (−0.19 vs. −0.07 cm^2^/year) [30]. Tian et al. have shown that PhenoAge acceleration predicts transitions to cardio-renal-metabolic multimorbidity [31]. Collectively, these mechanisms provide a mechanistic explanation for the observed association between BAA metrics and degenerative VHD risk.

Notably, no significant associations were observed for MR-related events. This likely reflects distinct pathophysiological mechanisms, as MR is frequently functional or secondary to left ventricular remodeling and cardiomyopathies rather than a primary degenerative leaflet process [32,33]. Therefore, systemic BAA may play a less direct role in driving clinically significant MR-related outcomes. Importantly, BAA may be reversible, as shown by a recent study in which biological age decelerated after stress recovery [34]. Risk factors such as hypertension, adiposity, dyslipidemia, hyperglycemia, smoking, and insufficient physical activity have been shown to accelerate aging-related epigenetic modifications [24]. Thus, interventions targeting metabolism, lifestyle, and physical activity may offer novel approaches to reducing the risk of degenerative VHD by reversing BA processes.

However, several limitations warrant consideration. First, residual confounding from unmeasured variables may persist despite comprehensive adjustment. Second, single-time biomarker assessment may not reflect the dynamic trajectory of BA or evolving changes in comorbidity treatment and adherence to therapy. Temporal variations in BAA and unmeasured time-varying confounders could have attenuated the true associations. Third, the UK Biobank’s demographic skew toward White, middle-aged individuals limits generalizability to other ethnicities and younger age groups. Fourth, ICD-code-based ascertainment of valvular severity risks under-detection of subclinical or asymptomatic lesions. Fifth, the UK Biobank cohort’s intrinsic healthy-volunteer bias may inadequately capture high-risk groups [35]. Notably, the robust prognostic utility of BAA is evident even within this comparatively low-risk cohort. Finally, although BAA robustly predicts incident disease, prospective interventional trials are required to establish whether attenuating these aging metrics prevents degenerative VHD.

In conclusion, we demonstrate that increased BAA (as quantified by both PhenoAge acceleration and KDM-BA acceleration) is associated with an increased risk of incident degenerative VHD and related adverse events, except for MR-related events. Given its effectiveness and accessibility, BAA may serve as a practical biomarker to guide prevention and personalized management of degenerative VHD.

## CRediT authorship contribution statement

**Chaoyang Lin:** Writing – review & editing, Writing – original draft, Visualization, Validation, Supervision, Software, Methodology, Investigation, Formal analysis, Conceptualization. **Enhao Wei:** Writing – review & editing, Writing – original draft, Methodology, Formal analysis. **Qianyao Lai:** Writing – review & editing, Writing – original draft, Software, Methodology, Formal analysis. **Hangpan Jiang:** Writing – review & editing, Writing – original draft. **Maosen Lin:** Writing – review & editing, Writing – original draft. **Feng Hu:** Writing – review & editing, Writing – original draft, Conceptualization. **Lin Fan:** Writing – review & editing, Writing – original draft, Conceptualization. **Enhui Yao:** Writing – review & editing, Writing – original draft, Funding acquisition, Conceptualization.

## Funding

This study was supported by the Fujian Province Traditional Chinese Medicine Heart Disease Key Discipline Construction Project (2024-363), the Cardiovascular Medicine Center of Fujian Province (0802701022100201), the Fujian Provincial Cardiovascular and Vascular Clinical Research Center (2021GPT0032021), and the Medical Innovation Project of Fujian Province (2018-CX-20).

## Declaration of competing interest

The authors declare that they have no known competing financial interests or personal relationships that could have appeared to influence the work reported in this paper.

## Data Availability

This study was conducted using data from the UK Biobank under Application Number 216062. The UK Biobank data can be accessed by researchers on application (https://www.ukbiobank.ac.uk/use-our-data/apply-for-access/).
