# Supplementary material for: Accelerated biological aging and incident degenerative valvular heart disease: Findings from 408,783 UK Biobank participants
Source: Int J Cardiol Heart Vasc. 2025 Nov 14;61:101838. doi: 10.1016/j.ijcha.2025.101838 (PMC12662120; doi:10.1016/j.ijcha.2025.101838)
Supplement: Supplementary Data 1 [file mmc1.docx]

**Supplementary Material**

**Table of contents**

[Table S1. STROBE checklist of items. 3](#_Toc209538323)

[Table S2. Ascertainment of outcomes. 9](#_Toc209538324)

[Table S3. Ascertainment of covariates of interest. 10](#_Toc209538325)

[Table S4. Incidence metrics and hazard ratios for aortic and mitral valve regurgitation related events across biological age accelerations quartiles (Primary Cohort; Model 1). 17](#_Toc209538326)

[Table S5. Subgroup analyses: Adjusted hazard ratio for degenerative valvular heart disease associated with biological age acceleration (Primary Cohort; Model 1). 19](#_Toc209538327)

[Table S6. Multicollinearity diagnostics of risk factors in Primary Cohort. 24](#_Toc209538328)

[Figure S1. Adjusted survival curves and dose–response relationships between biological age accelerations and the risk of aortic valve regurgitation-related and mitral valve regurgitation-related events. 25](#_Toc209538329)

[Figure S2. Adjusted hazard ratio for degenerative valvular heart disease across BAAs quartiles (Primary Cohort; Model 2). 27](#_Toc209538330)

[Figure S3. Adjusted hazard ratio for degenerative valvular heart disease across BAAs quartiles excluding participants with baseline cardiovascular comorbidities (coronary artery disease, heart failure, atrial fibrillation, cardiomyopathy, and chronic kidney disease) (Analytic Cohort 2; Model 2). 29](#_Toc209538331)

[Figure S4. Adjusted hazard ratio for degenerative valvular heart disease across BAAs quartiles excluding participants with baseline cardiovascular comorbidities and with follow-up duration <2 years. (Analytic Cohort 3; Model 2) 31](#_Toc209538332)

## Table S1. STROBE checklist of items.

|  | **Item No.** | **Recommendation** | **Page/Section Reference** | **Relevant text from manuscript** |
| --- | --- | --- | --- | --- |
| **Title and abstract** | 1 | (*a*) Indicate the study’s design with a commonly used term in the title or the abstract | Title & Abstract | \|  \| \| --- \|  \| Title: “Accelerated biological aging and incident degenerative valvular heart disease: Findings from 408,783 UK Biobank participants” Abstract: “This prospective cohort included 408,783 UK Biobank participants...” \| \| --- \| |
|  |  | (*b*) Provide in the abstract an informative and balanced summary of what was done and what was found | Abstract | Clear summary of background, exposure (BAAs), outcomes (incident VHD and events), methods, results, and conclusions. |
| **Introduction** | | | |  |
| Background/rationale | 2 | Explain the scientific background and rationale for the investigation being reported | Introduction | “The aging population is increasingly burdened by degenerative VHD... BAAs have been linked to cardiovascular diseases, but associations with VHD are unclear.” |
| Objectives | 3 | State specific objectives, including any prespecified hypotheses Introduction | Introduction | "This study aimed to investigate the dose-response relationship between biological age acceleration (BAA) and degenerative VHD risk among middle-aged adults." |
| **Methods** | | | |  |
| Study design | 4 | Present key elements of study design early in the paper | Methods – Study Population | “This prospective cohort included 408,783 UK Biobank participants without baseline valvular disease for primary time-to-event analysis.” |
| Setting | 5 | Describe the setting, locations, and relevant dates, including periods of recruitment, exposure, follow-up, and data collection | Methods – Study Population | “UK Biobank recruited participants aged 37–73 years (2006–2010)... Median follow-up 13.91 years.” |
| Participants | 6 | (*a*) *Cohort study*—Give the eligibility criteria, and the sources and methods of selection of participants. Describe methods of follow-up  *Case-control study*—Give the eligibility criteria, and the sources and methods of case ascertainment and control selection. Give the rationale for the choice of cases and controls  *Cross-sectional study*—Give the eligibility criteria, and the sources and methods of selection of participants | Methods – Study Population | Excluded: missing BAA traits, covariates, baseline VHD, loss to follow-up. Used follow-up time as timescale. |
|  |  | (*b*) *Cohort study*—For matched studies, give matching criteria and number of exposed and unexposed  *Case-control study*—For matched studies, give matching criteria and the number of controls per case | N/A | Not applicable |
| Variables | 7 | Clearly define all outcomes, exposures, predictors, potential confounders, and effect modifiers. Give diagnostic criteria, if applicable | Methods – Outcomes/Covariates | Outcomes: AS, AR, MR, events. Exposures: PhenoAge/KDM-BAA. Confounders: demographics, lifestyle, clinical. |
| Data sources/ measurement | 8* | For each variable of interest, give sources of data and details of methods of assessment (measurement). Describe comparability of assessment methods if there is more than one group | Methods – Assessment sections | BAAs: BioAge R package; outcomes: ICD-10 codes; validated in prior studies. |
| Bias | 9 | Describe any efforts to address potential sources of bias | Methods & Discussion – Limitations | Sensitivity analyses (exclude comorbidities, short follow-up), VIF to check collinearity. |
| Study size | 10 | Explain how the study size was arrived at | N/A | Not explicitly calculated (used full eligible UKB sample). |

| Quantitative variables | 11 | Explain how quantitative variables were handled in the analyses. If applicable, describe which groupings were chosen and why | Methods – Statistical Analysis | Standardized BAAs (mean = 0, SD = 1); quartile categorization for dose-response. |
| --- | --- | --- | --- | --- |
| Statistical methods | 12 | (*a*) Describe all statistical methods, including those used to control for confounding | Methods – Statistical Analysis | Cox regression (HRs, 95% CI); Model 1 (basic); Model 2 (comorbidities/medications); Fine–Gray for events. |
|  |  | (*b*) Describe any methods used to examine subgroups and interactions | Methods – Statistical Analysis | Subgroup analyses across demographics and comorbidities. |
|  |  | (*c*) Explain how missing data were addressed | Methods – Study Population | Participants with missing data excluded. |
|  |  | (*d*) *Cohort study*—If applicable, explain how loss to follow-up was addressed  *Case-control study*—If applicable, explain how matching of cases and controls was addressed  *Cross-sectional study*—If applicable, describe analytical methods taking account of sampling strategy | \|  \| \| --- \|  \| Methods – Study Population \| \| --- \| | Loss to follow-up excluded from primary analysis. |
|  |  | (*e*) Describe any sensitivity analyses | Methods – Statistical Analysis | Excluded CVD at baseline; excluded follow-up <2 years. |
| **Results** | | | | |
| Participants | 13* | (a) Report numbers of individuals at each stage of study—eg numbers potentially eligible, examined for eligibility, confirmed eligible, included in the study, completing follow-up, and analysed | Methods – Study Population | 408,783 in primary analysis; 376,323 and 347,551 in sensitivity sets. Figure 1. |
|  |  | (b) Give reasons for non-participation at each stage | N/A | Not applicable (retrospective inclusion of existing data). |
|  |  | (c) Consider use of a flow diagram | Figure 1 | Included. |
| Descriptive data | 14* | (a) Give characteristics of study participants (eg demographic, clinical, social) and information on exposures and potential confounders | Table 1 | Baseline demographics, clinical, lifestyle, and biomarker characteristics. |
|  |  | (b) Indicate number of participants with missing data for each variable of interest | N/A | Not explicitly stated; missingness excluded during cohort derivation. |
|  |  | (c) *Cohort study*—Summarise follow-up time (eg, average and total amount) | Results | “Over a median follow-up of 13.91 years...” |
| Outcome data | 15* | *Cohort study*—Report numbers of outcome events or summary measures over time | Results | Reported total incident cases (AS, AR, MR, events) with rates. |
|  |  | *Case-control study—*Report numbers in each exposure category, or summary measures of exposure |  |  |
|  |  | *Cross-sectional study—*Report numbers of outcome events or summary measures |  |  |
| Main results | 16 | (*a*) Give unadjusted estimates and, if applicable, confounder-adjusted estimates and their precision (eg, 95% confidence interval). Make clear which confounders were adjusted for and why they were included | Tables 2–4, S4 | Adjusted HRs with 95% CIs reported; models explained. |
|  |  | (*b*) Report category boundaries when continuous variables were categorized | Results | Quartiles of BAA reported (Q1–Q4), HRs compared to Q1. |
|  |  | (*c*) If relevant, consider translating estimates of relative risk into absolute risk for a meaningful time period | Table 4 | 5-year and 10-year absolute risks by BAA strata. |

| Other analyses | 17 | Report other analyses done—eg analyses of subgroups and interactions, and sensitivity analyses | Results – Additional analysis | Subgroups: age, sex, lifestyle. Sensitivity: CVD exclusion, follow-up <2 years. |
| --- | --- | --- | --- | --- |
| **Discussion** | | | | |
| Key results | 18 | Summarise key results with reference to study objectives | Discussion | Summarizes AS, AR, MR, BAA associations. |
| Limitations | 19 | Discuss limitations of the study, taking into account sources of potential bias or imprecision. Discuss both direction and magnitude of any potential bias | Discussion – Limitations | Discusses confounding, generalizability, single-point BA, coding issues. |
| Interpretation | 20 | Give a cautious overall interpretation of results considering objectives, limitations, multiplicity of analyses, results from similar studies, and other relevant evidence | Discussion | Interprets findings in context of literature and biological mechanisms. |
| Generalisability | 21 | Discuss the generalisability (external validity) of the study results | Discussion: Limitations | Discussed UKB population limits: White, middle-aged, healthy volunteer bias. |
| **Other information** | |  | | |
| Funding | 22 | Give the source of funding and the role of the funders for the present study and, if applicable, for the original study on which the present article is based | Declarations: Funding | Lists all sources of funding and grant IDs; role not specified, no conflict. |

*Give information separately for cases and controls in case-control studies and, if applicable, for exposed and unexposed groups in cohort and cross-sectional studies.

**Note:** An Explanation and Elaboration article discusses each checklist item and gives methodological background and published examples of transparent reporting. The STROBE checklist is best used in conjunction with this article (freely available on the Web sites of PLoS Medicine at http://www.plosmedicine.org/, Annals of Internal Medicine at http://www.annals.org/, and Epidemiology at http://www.epidem.com/). Information on the STROBE Initiative is available at www.strobe-statement.org.

## Table S2. Ascertainment of outcomes.

| **Source** | **UKB Field ID or ICD code** | **Description** |
| --- | --- | --- |
| Aortic valve stenosis | Self-report (20002) |  |
|  | 1490 | Aortic stenosis |
|  | Diagnoses - ICD10 (41270) |  |
|  | I350 | Aortic (valve) stenosis |
|  | I352 | Aortic (valve) stenosis with insufficiency |
| Aortic valve regurgitation | Self-report (20002) |  |
|  | 1587 | Aortic regurgitation / incompetence |
|  | Diagnoses - ICD10 (41270) |  |
|  | I351 | Aortic (valve) insufficiency |
| Aortic valve regurgitation | Self-report (20002) |  |
|  | 1585 | Mitral regurgitation / incompetence |
|  | Diagnoses - ICD10 (41270) |  |
|  | I340 | Mitral (valve) insufficiency |
| Aortic valve regurgitation related intervention or mortality | Diagnoses - ICD10 (41270) | [I350 (Diagnoses) & K26 (OPCS4)] or  [I352 (Diagnoses) & K26 (OPCS4)] or  I350 (Death register) or I352 (Death register) |
|  | Operative procedures (41272) |  |
|  | Death register (40001, 40002) |  |
| Aortic valve regurgitation related intervention or mortality | Diagnoses - ICD10 (41270) | [I351 (Diagnoses) & K26 (OPCS4)] or  I351 (Death register) |
|  | Operative procedures (41272) |  |
|  | Death register (40001, 40002) |  |
| Mitral valve regurgitation related intervention or mortality | Diagnoses - ICD10 (41270) | [I340 (Diagnoses) & K25/K341 (OPCS4)] or  I340 (Death register) |
|  | Operative procedures (41272) |  |
|  | Death register (40001, 40002) |  |

## Table S3. Ascertainment of covariates of interest.

| **Source** | **UKB Field ID or ICD code** | **Description** |
| --- | --- | --- |
| **Demographics** |  |  |
| Age | 21003 |  |
| Sex | 31 |  |
| Race (white) | 21000 |  |
| Education (college) | 6138 |  |
| Body mass index | 23104, 21001 |  |
| Smoking status | 20116 |  |
| Alcohol intake frequency | 1558 |  |
| Healthy physical activity | 100054 | Physical activity |
| Townsend deprivation score | 22189 |  |
| **Comorbidities** |  |  |
| Hypertension | Self-report (20002) |  |
|  | 1065 | Hypertension |
|  | 1072 | Essential hypertension |
|  | Diagnosed by doctor |  |
|  | 6150: 4 | High blood pressure |
|  | 2966 | Age high blood pressure diagnosed |
|  | Diagnoses - ICD 9 (41271) |  |
|  | 401-405 |  |
|  | Diagnoses - ICD10 (41270) |  |
|  | I10 | Essential (primary) hypertension |
|  | I11 | Hypertensive heart disease |
|  | I12 | Hypertensive renal disease |
|  | I13 | Hypertensive heart and renal disease |
|  | I15 | Secondary hypertension |
|  | First occurrences |  |
|  | 131286, 131288, 131290, 131292, 131294 | I10, I11, I12, I13, I15 |
|  | GP Clinical records (42040) |  |
|  | ctv | .14A2, .6627, .6628, .662d, .662F, .662O, .662P, .G3.., .G31.,  .G35., .G36., 14A2., 662d., 662F., 662O., 662P., 662P0, G200.,  G201., G203., G24.., G240., G24z., G24zz, G26.., G28.., Xa3fQ,  Xa8HD, XaIyE, XaXOi, XaZbz, XaZWn, XE0Ub, XE0Uc, XE0Ud, XE0W8, XM1YA, XSDSb |
|  | Antihypertensive medication |  |
| Obesity | First occurrences: 130792 | E66, Obesity |
| Dyslipidemia | Self-reported 20002: 1473 | High cholesterol |
|  | Diagnoses - ICD 9 (41271) |  |
|  | Diagnoses - ICD10 (41270) |  |
|  | E780 | Pure hypercholesterolaemia |
|  | E781 | Pure hyperglyceridaemia |
|  | E782 | Mixed hyperlipidaemia |
|  | E783 | Hyperchylomicronaemia |
|  | E784 | Other hyperlipidaemia |
|  | E785 | Hyperlipidaemia, unspecified |
|  | First occurrences |  |
|  | 130814 | E78, disorders of lipoprotein metabolism and other lipidaemias |
|  | Lipid-lowering medication |  |
| Diabetes | Self-report (20002) |  |
|  | 1220 | Diabetes |
|  | 1222 | Type 1 diabetes |
|  | 1223 | Type 2 diabetes |
|  | Diagnosed by doctor |  |
|  | 2443 | Diabetes diagnosed by doctor |
|  | 2976 | Age diabetes diagnosed by doctor |
|  | Diagnoses - ICD 9 (41271): 250 |  |
|  | Diagnoses - ICD10 (41270) |  |
|  | E10 | Type 1 diabetes mellitus |
|  | E11 | Type 2 diabetes mellitus |
|  | E13 | Other specified diabetes mellitus |
|  | E14 | Unspecified diabetes mellitus |
|  | G590 | Diabetic mononeuropathy |
|  | G632 | Diabetic polyneuropathy |
|  | H280 | Diabetic cataract |
|  | H360 | Diabetic retinopathy |
|  | M142 | Diabetic arthropathy |
|  | N083 | Glomerular disorders in diabetes mellitus |
|  | O240 | Diabetes mellitus in pregnancy: Pre-existing type 1 diabetes mellitus |
|  | O241 | Diabetes mellitus in pregnancy: Pre-existing type 2 diabetes mellitus |
|  | O243 | Diabetes mellitus in pregnancy: Pre-existing diabetes mellitus, unspecified |
|  | O244 | Diabetes mellitus arising in pregnancy |
|  | O249 | Diabetes mellitus in pregnancy, unspecified |
|  | Y423 | Insulin and oral hypoglycaemic [antidiabetic] drugs |
|  | First occurrences 130706, 130708, | E10, E11, E13, E14 |
|  | 130712, 130714 |  |
|  | HbA1c (30750, Glycated haemoglobin) | ≥6.5% |
|  | Anti-diabetic medication | As below |
| Osteoporosis | First occurrences |  |
|  | 131962 | M80, Osteoporosis with pathological fracture |
|  | 131964 | M81, Osteoporosis without pathological fracture |
|  | 131966 | M82, Osteoporosis in diseases classified elsewhere |
| Coronary artery disease | Self-report (20002) |  |
|  | 1074 | Angina |
|  | 1075 | Heart attack/myocardial infarction |
|  | Diagnosed by doctor |  |
|  | 6150: 2, 1 | Heart attack, Angina |
|  | 3627 | Age angina diagnosed |
|  | 2976 | Age heart attack diagnosed |
|  | Diagnoses - ICD 9 (41271) |  |
|  | 410-414 |  |
|  | Diagnoses - ICD10 (41270) |  |
|  | I20 | Angina pectoris |
|  | I21 | Acute myocardial infarction |
|  | I22 | Subsequent myocardial infarction |
|  | I23 | Certain current complications following acute myocardial infarction |
|  | I24 | Other acute ischaemic heart diseases |
|  | I25 | Chronic ischaemic heart disease |
|  | First occurrences |  |
|  | 131296, 131298, 131300, 131302,  131304, 131306 | I20, I21, I22, I23, I24, I25 |
| Heart failure | Self-report (20002): 1076 | Heart failure/pulmonary odema |
|  | Diagnoses - ICD 9 (41271): 428 | Heart failure |
|  | Diagnoses - ICD10 (41270): I50 | Heart failure |
|  | First occurrences: 131354 | I50, heart failure |
| Atrial fibrillation | Self-report (20002) |  |
|  | 1471 | Atrial fibrillation |
|  | 1483 | Atrial flutter |
|  | Diagnoses - ICD 9 (41271) |  |
|  | 4273 | Atrial fibrillation and flutter |
|  | Diagnoses - ICD10 (41270) |  |
|  | I48 | Atrial fibrillation and flutter |
|  | First occurrences |  |
|  | 131350 | I48, Atrial fibrillation and flutter |
| Cardiomyopathy | Self-report (20002) |  |
|  | 1079 | Cardiomyopathy |
|  | 1588 | Hypertrophic cardiomyopathy (hcm / hocm) |
|  | Diagnoses - ICD 9 (41271) |  |
|  | 425 | Cardiomyopathy |
|  | Diagnoses - ICD10 (41270) |  |
|  | I42 | Cardiomyopathy |
|  | I43 | Cardiomyopathy in diseases classified elsewhere |
|  | First occurrences |  |
|  | 131338 | Cardiomyopathy |
|  | 131340 | Cardiomyopathy in diseases classified elsewhere |
| Chronic kidney disease | First occurrences: 132032 | N18, Chronic renal failure |
| **Medications** |  |  |
| Antihypertensive medication | Medications (6153, 6177): 2 | Blood pressure medication |
|  | Self-reported medications (20003) | 1140860334, 1140860336, 1140860338, 1140860340, 1140860342, 1140860348, 1140860352,  1140860356, 1140860358, 1140860380, 1140860382, 1140860386, 1140860390, 1140860394,  1140860396, 1140860398, 1140860402, 1140860404, 1140860406, 1140860410, 1140860418,  1140860422, 1140860426, 1140860434, 1140860492, 1140860498, 1140860562, 1140860564,  1140860580, 1140860590, 1140860610, 1140860654, 1140860658, 1140860690, 1140860696,  1140860706, 1140860714, 1140860728, 1140860736, 1140860738, 1140860750, 1140860752,  1140860758, 1140860764, 1140860776, 1140860784, 1140860790, 1140860802, 1140860806,  1140860878, 1140860882, 1140860892, 1140860904, 1140860912, 1140860918, 1140861088,  1140861090, 1140861106, 1140861110, 1140861114, 1140861120, 1140861128, 1140861130,  1140861136, 1140861138, 1140861166, 1140861176, 1140861190, 1140861194, 1140861202,  1140861276, 1140861282, 1140864950, 1140864952, 1140866072, 1140866078, 1140866090,  1140866092, 1140866094, 1140866096, 1140866102, 1140866104, 1140866122, 1140866128,  1140866132, 1140866136, 1140866138, 1140866140, 1140866144, 1140866146, 1140866156,  1140866158, 1140866162, 1140866164, 1140866168, 1140866226, 1140866232, 1140866236,  1140866244, 1140866262, 1140866306, 1140866308, 1140866312, 1140866318, 1140866324,  1140866328, 1140866330, 1140866340, 1140866352, 1140866354, 1140866360, 1140866396,  1140866400, 1140866402, 1140866404, 1140866410, 1140866416, 1140866420, 1140866440,  1140866446, 1140866450, 1140866460, 1140866466, 1140866484, 1140866546, 1140866554,  1140866692, 1140866704, 1140866712, 1140866724, 1140866726, 1140866738, 1140866756,  1140866758, 1140866764, 1140866766, 1140866778, 1140866782, 1140866784, 1140866798,  1140866800, 1140866802, 1140866804, 1140879760, 1140879762, 1140879778, 1140879782,  1140879786, 1140879794, 1140879798, 1140879802, 1140879806, 1140879810, 1140879818,  1140879824, 1140879830, 1140879834, 1140879842, 1140879866, 1140888510, 1140888552,  1140888556, 1140888560, 1140888578, 1140888646, 1140909368, 1140911698, 1140916356,  1140916362, 1140917428, 1140923572, 1140923712, 1140923718, 1140926778, 1140926780,  1141145658, 1141145660, 1141145668, 1141151016, 1141151018, 1141151382, 1141152600,  1141152998, 1141153006, 1141153026, 1141153032, 1141153328, 1141156754, 1141156808,  1141156836, 1141156846, 1141164148, 1141164154, 1141164276, 1141164280, 1141165470,  1141165476, 1141166006, 1141167822, 1141167832, 1141171152, 1141171336, 1141171344,  1141172682, 1141172686, 1141180592, 1141180598, 1141187788, 1141187790, 1141190160,  1141193282, 1141193346, 1141194794, 1141194800, 1141194804, 1141194808, 1141194810,  1141201038, 1141201040 |
| Lipid-lowering medication | Medications (6153, 6177): 1 | Cholesterol lowering medication |
|  | Self-reported medications (20003) | 1141146138, 1141146234, 1141192414, 1140861958, 1140881748, 1141200040, 1140888648,  1141192410, 1140864592, 1140888594, 1140861954, 1140861924, 1141157260, 1140862026,  1140861944, 1140862026, 1140861928, 1140861926, 1141162544 |
| Anti-diabetic medication | Medications (6153, 6177): 3 | Insulin |
|  | Self-reported medications (20003) | 1140884600, 1140874744, 1141152590, 1140874646, 1140874674, 1141171646, 1140883066,  1141168660 |
| Antithrombotic medication | Self-reported medications (20003) | Antiplatelet: 1140861776, 1140868226, 1140861806, 1140864860, 1140868282, 1140872040,  1140882190, 1140882268, 1140882392, 1141163138, 1141164044, 1141167844, 1140861808,  1140882192, 1140909772, 1140911754, 1140909480, 1140856336, 1140917114, 1140856412,  1140861780, 1140861804, 1140868258, 1140882108, 1140909890, 1141167848, 1141168318,  1141168322, 1140861800, 1141163324, 1140861778, 1140909712 |
|  |  | Anticoagulation: 11409108+B132:D13932,1140888266,1141164760, 1140881842, 1140864956,  1140861588, 140888204, 1140888206, 1140864212, 1141189054, 1140926444, 1140861696,  1140864122, 1140861698, 1140861702 1140909770 |
| **Valvular heart diseases** |  |  |
| Rheumatic valve disease |  |  |
| Mitral valve | First occurrences: 131276 | I05, Rheumatic mitral valve diseases |
| Aortic valve | First occurrences: 131278 | I06, Rheumatic aortic valve diseases |
| Tricuspid valve | First occurrences: 131280 | I07, Rheumatic tricuspid valve diseases |
| Congenital valve disease or Marfan's syndrome | |  |
| Pulmonary and tricuspid valves | First occurrences: 132470 | Q22, Congenital malformations of pulmonary and tricuspid valves |
| Aortic and mitral valves | First occurrences: 132472 | Q23, Congenital malformations of aortic and mitral valves |
| Marfan's syndrome | Diagnoses - ICD10 (41270) | Q874, Marfan's syndrome |
| Endocarditis with valvular heart disease |  |  |
| Valve unspecified | First occurrences: 131330 | I38, Endocarditis, valve unspecified |
| Heart valve disorders in diseases  classified elsewhere | First occurrences: 131332 | I39, Endocarditis and heart valve disorders in diseases classified elsewhere |
| Nonrheumatic heart diseases |  |  |
| Mitral valve disorders | First occurrences: 131322 | I34, Nonrheumatic mitral valve disorders |
| Aortic valve disorders | First occurrences: 131324 | I35, Nonrheumatic aortic valve disorders |
| Tricuspid valve disorders | First occurrences: 131326 | I36, Nonrheumatic tricuspid valve disorders |
| Pulmonary valve disorders | First occurrences: 131328 | I37, Pulmonary valve disorders |
| Multiple valve disorders | First occurrences: 131282 | I08, Multiple valve diseases |

Note that three-digit codes encompass all corresponding four-digit subcodes, such as E10 (E100–E102). ICD, international classification disease; OPCS, Office of Population Censuses and Surveys Classification of Interventions and Procedures. GP, general practice.

## Table S4. Incidence metrics and hazard ratios for aortic and mitral valve regurgitation related events across biological age accelerations quartiles (Primary Cohort; Model 1).

|  |  | **PhenoAge acceleration Quartiles** | | | | |
| --- | --- | --- | --- | --- | --- | --- |
|  |  | **Total** | **Q1** | **Q2** | **Q3** | **Q4** |
| Aortic valve regurgitation related intervention or mortality | Event, n | 244 (0.06%) | 39 (0.04%) | 47 (0.05%) | 76 (0.07%) | 82 (0.08%) |
|  | Incidence rate ^a^ | 0.47 (0.41, 0.53) | 0.29 (0.21, 0.40) | 0.35 (0.27, 0.47) | 0.58 (0.46, 0.73) | 0.69 (0.55, 0.85) |
|  | Adjusted incidence rate ^a,b^ | - | 0.29 (0.19, 0.44) | 0.29 (0.2, 0.44) | 0.43 (0.3, 0.62) | 0.47 (0.33, 0.66) |
|  | Adjusted incidence rate difference ^a,b^ | - | ref | 0.00 (-0.15, 0.15) | **0.18 (0.04, 0.35)** | **0.22 (0.04, 0.36)** |
|  | Adjusted incidence rate ratio ^b^ | - | ref | 1.01 (0.68, 1.61) | **1.50 (1.08, 2.34)** | **1.62 (1.09, 2.31)** |
|  | Adjusted hazard ratio ^b^ | - | ref | 1.01 (0.66, 1.54) | **1.48 (1.001, 2.19)** | **1.60 (1.08, 2.39)** |
| Mitral valve regurgitation related intervention or mortality | Event, n | 510 (0.12%) | 100 (0.10%) | 132 (0.13%) | 139 (0.14%) | 139 (0.14%) |
|  | Incidence rate ^a^ | 0.98 (0.90, 1.07) | 0.74 (0.61, 0.90) | 0.99 (0.84, 1.18) | 1.06 (0.90, 1.26) | 1.16 (0.98, 1.37) |
|  | Adjusted incidence rate ^a,b^ | - | 0.54 (0.41, 0.72) | 0.67 (0.51, 0.87) | 0.67 (0.52, 0.88) | 0.71 (0.54, 0.92) |
|  | Adjusted incidence rate difference ^a,b^ | - | ref | 0.19 (-0.001, 0.39) | **0.21 (0.03, 0.41)** | **0.26 (0.01, 0.47)** |
|  | Adjusted incidence rate ratio ^b^ | - | ref | **1.24 (1.00, 1.53)** | **1.26 (1.03, 1.53)** | **1.32 (1.01, 1.66)** |
|  | Adjusted hazard ratio ^b^ | - | ref | 1.23 (0.95, 1.60) | 1.24 (0.96, 1.62) | 1.30 (0.99, 1.71) |

|  |  | **KDM-BA acceleration Quartiles** | | | | |
| --- | --- | --- | --- | --- | --- | --- |
|  |  | **Total** | **Q1** | **Q2** | **Q3** | **Q4** |
| Aortic valve regurgitation related intervention or mortality | Event, n | 244 (0.06%) | 60 (0.06%) | 52 (0.05%) | 63 (0.06%) | 69 (0.07%) |
|  | Incidence rate ^a^ | 0.47 (0.41, 0.53) | 0.46 (0.36, 0.60) | 0.39 (0.30, 0.51) | 0.48 (0.38, 0.61) | 0.55 (0.43, 0.70) |
|  | Adjusted incidence rate ^a,b^ | - | 0.30 (0.20, 0.43) | 0.31 (0.21, 0.46) | 0.41 (0.28, 0.59) | 0.47 (0.33, 0.66) |
|  | Adjusted incidence rate difference ^a,b^ | - | ref | 0.02 (-0.11, 0.16) | 0.14 (-0.003, 0.30) | **0.22 (0.09, 0.41)** |
|  | Adjusted incidence rate ratio ^b^ | - | ref | 1.05 (0.75, 1.52) | 1.37 (0.99, 1.93) | **1.58 (1.23, 2.31)** |
|  | Adjusted hazard ratio ^b^ | - | ref | 1.05 (0.73, 1.52) | 1.38 (0.96, 1.97) | **1.58 (1.10, 2.26)** |
| Mitral valve regurgitation related intervention or mortality | Event, n | 510 (0.12%) | 136 (0.13%) | 122 (0.12%) | 136 (0.13%) | 116 (0.11%) |
|  | Incidence rate ^a^ | 0.98 (0.90, 1.07) | 1.05 (0.89, 1.24) | 0.92 (0.77, 1.10) | 1.04 (0.88, 1.23) | 0.93 (0.77, 1.11) |
|  | Adjusted incidence rate ^a,b^ | - | 0.61 (0.47, 0.80) | 0.63 (0.48, 0.82) | 0.72 (0.55, 0.94) | 0.64 (0.49, 0.84) |
|  | Adjusted incidence rate difference ^a,b^ | - | ref | 0.02 (-0.18, 0.19) | 0.16 (-0.07, 0.34) | 0.04 (-0.23, 0.28) |
|  | Adjusted incidence rate ratio ^b^ | - | ref | 1.02 (0.82, 1.23) | 1.17 (0.93, 1.42) | 1.04 (0.78, 1.33) |
|  | Adjusted hazard ratio ^b^ | - | ref | 1.02 (0.80, 1.30) | 1.17 (0.92, 1.49) | 1.04 (0.81, 1.35) |

^a^ Per 10,000 person-years (95% confidence interval).
^b^ Adjusted for age, sex, ethnicity, education, smoking status, alcohol intake frequency, healthy physical activity, and Townsend deprivation index.
Adjusted incidence rate difference, adjusted incidence rate ratio, and adjusted hazard ratio in bold represent statistical significance (*P*<0.05).

## Table S5. Subgroup analyses: Adjusted hazard ratio for degenerative valvular heart disease associated with biological age acceleration (Primary Cohort; Model 1).

| **Aortic valve stenosis** | | | | | | | |
| --- | --- | --- | --- | --- | --- | --- | --- |
| **Subgroup Analysis** |  | **PhenoAge acceleration** | | | **KDM-BA acceleration** | | |
|  | **Events, n(%)** | **HR (95% CI)** | **P-value** | **P-interaction** | **HR (95% CI)** | **P-value** | **P-interaction** |
| Chronological age (years) |  |  |  |  |  |  |  |
| ＜65 | 2,425 (0.73%) | 1.33 (1.29 ,1.36) | <0.001 | 0.153 | 1.44 (1.39, 1.50) | <0.001 | <0.001 |
| ≥65 | 2,177 (2.8%) | 1.32 (1.28, 1.36) | <0.001 |  | 1.23 (1.19, 1.28) | <0.001 |  |
| Sex |  |  |  |  |  |  |  |
| Women | 1,682 (0.76%) | 1.35 (1.31, 1.40) | <0.001 | 0.017 | 1.35 (1.28, 1.42) | <0.001 | 0.301 |
| Men | 2,920 (1.55%) | 1.31 (1.28, 1.34) | <0.001 |  | 1.33 (1.28, 1.37) | <0.001 |  |
| College educated |  |  |  |  |  |  |  |
| No | 3,538 (1.28%) | 1.33 (1.30, 1.36) | <0.001 | 0.047 | 1.31 (1.27, 1.36) | <0.001 | 0.215 |
| Yes | 1,064 (0.8%) | 1.27 (1.21, 1.34) | <0.001 |  | 1.39 (1.31, 1.47) | <0.001 |  |
| Smoking status, n (%) |  |  |  |  |  |  |  |
| Never | 1,880 (0.48%) | 1.32 (1.28, 1.36) | <0.001 | 0.688 | 1.40 (1.34, 1.46) | <0.001 | 0.017 |
| Previous or Current | 2,722 (0.57%) | 1.33 (1.29, 1.36) | <0.001 |  | 1.30 (1.25, 1.34) | <0.001 |  |
| Alcohol intake frequency, n (%) | |  |  |  |  |  |  |
| Less than daily | 3,563 (0.33%) | 1.33 (1.30, 1.36) | <0.001 | 0.766 | 1.33 (1.29, 1.39) | <0.001 | 0.968 |
| Daily |  | 1.32 (1.26, 1.38) | <0.001 |  | 1.33 (1.28, 1.39) | <0.001 |  |
| Healthy physical activity |  |  |  |  |  |  |  |
| No | 2,280 (1.22%) | 1.33 (1.30, 1.37) | <0.001 | 0.438 | 1.33 (1.29, 1.39) | <0.001 | 0.415 |
| Yes | 2,322 (1.05%) | 1.31 (1.27, 1.35) | <0.001 |  | 1.33 (1.28, 1.39) | <0.001 |  |
| Hypertension |  |  |  |  |  |  |  |
| No | 1,669 (0.62%) | 1.23 (1.18, 1.29) | <0.001 | 0.030 | 1.34 (1.28, 1.41) | <0.001 | 0.105 |
| Yes | 2,933 (2.10%) | 1.30 (1.27, 1.33) | <0.001 |  | 1.26 (1.22, 1.30) | <0.001 |  |
| Dyslipidemia |  |  |  |  |  |  |  |
| No | 1,805 (0.62%) | 1.25 (1.20, 1.30) | <0.001 | 0.174 | 1.38 (1.32, 1.45) | <0.001 | 0.009 |
| Yes | 2,797 (2.37%) | 1.30 (1.27, 1.33) | <0.001 |  | 1.27 (1.23, 1.31) | <0.001 |  |
| Diabetes |  |  |  |  |  |  |  |
| No | 3,741 (0.98%) | 1.28 (1.25, 1.31) | <0.001 | 0.395 | 1.31 (1.27, 1.35) | <0.001 | 0.226 |
| Yes | 861 (3.37%) | 1.25 (1.20, 1.30) | <0.001 |  | 1.27 (1.23, 1.31) | <0.001 |  |
| BMI |  |  |  |  |  |  |  |
| <30 kg/m2 | 2,673 (0.87%) | 1.29 (1.25, 1.33) | <0.001 | 0.964 | 1.35 (1.30, 1.40) | <0.001 | 0.002 |
| ≥30 kg/m2 | 1,914 (1.91%) | 1.30 (1.26, 1.34) | <0.001 |  | 1.21 (1.16, 1.26) | <0.001 |  |

| **Aortic valve stenosis-related intervention or mortality** | | | | | | | |
| --- | --- | --- | --- | --- | --- | --- | --- |
| **Subgroup Analysis** |  | **PhenoAge acceleration** | | | **KDM-BA acceleration** | | |
|  | **Events, n(%)** | **HR (95% CI)** | **P-value** | **P-interaction** | **HR (95% CI)** | **P-value** | **P-interaction** |
| Chronological age (years) |  |  |  |  |  |  |  |
| ＜65 | 961 (0.29%) | 1.27 (1.20, 1.33) | <0.001 | 0.547 | 1.43 (1.34, 1.52) | <0.001 | 0.090 |
| ≥65 | 717 (0.92%) | 1.31 (1.23, 1.39) | <0.001 |  | 1.29 (1.20, 1.38) | <0.001 |  |
| Sex |  |  |  |  |  |  |  |
| Women | 538(0.24%) | 1.30 (1.22, 1.39) | <0.001 | 0.437 | 1.45 (1.32, 1.60) | <0.001 | 0.108 |
| Men | 1,140 (0.60%) | 1.27 (1.22, 1.34) | <0.001 |  | 1.34 (1.27, 1.41) | <0.001 |  |
| College educated |  |  |  |  |  |  |  |
| No | 1,231 (0.45%) | 1.29 (1.24, 1.35) | <0.001 | 0.113 | 1.33 (1.26, 1.41) | <0.001 | 0.197 |
| Yes | 447 (0.34%) | 1.22 (1.12, 1.34) | <0.001 |  | 1.45 (1.32, 1.59) | <0.001 |  |
| Smoking status, n (%) |  |  |  |  |  |  |  |
| Never | 721 (0.32%) | 1.26 (1.19, 1.34) | <0.001 | 0.518 | 1.43 (1.33, 1.54) | <0.001 | 0.208 |
| Previous or Current | 957 (0.52%) | 1.29 (1.23, 1.36) | <0.001 |  | 1.32 (1.25, 1.41) | <0.001 |  |
| Alcohol intake frequency, n (%) | |  |  |  |  |  |  |
| Less than daily | 1,274 (0.39%) | 1.29 (1.23, 1.34) | <0.001 | 0.566 | 1.35 (1.28, 1.43) | <0.001 | 0.653 |
| Daily | 404 (0.48%) | 1.25 (1.14, 1.36) | <0.001 |  | 1.40 (1.28, 1.54) | <0.001 |  |
| Healthy physical activity |  |  |  |  |  |  |  |
| No | 761 (0.41%) | 1.30 (1.23, 1.36) | <0.001 | 0.558 | 1.35 (1.26, 1.44) | <0.001 | 0.789 |
| Yes | 917 (0.41%) | 1.26 (1.19, 1.34) | <0.001 |  | 1.38 (1.29, 1.47) | <0.001 |  |
| Hypertension |  |  |  |  |  |  |  |
| No | 680 (0.25%) | 1.15 (1.06, 1.25) | 0.001 | 0.031 | 1.37 (1.27, 1.49) | <0.001 | 0.343 |
| Yes | 998 (0.71%) | 1.29 (1.24, 1.35) | <0.001 |  | 1.29 (1.22, 1.37) | <0.001 |  |
| Dyslipidemia |  |  |  |  |  |  |  |
| No | 730 (0.25%) | 1.15 (1.07, 1.24) | <0.001 | 0.018 | 1.39 (1.29, 1.50) | <0.001 | 0.351 |
| Yes | 948 (0.80%) | 1.29 (1.24, 1.35) | <0.001 |  | 1.31 (1.23, 1.39) | <0.001 |  |
| Diabetes |  |  |  |  |  |  |  |
| No | 1,410 (0.37%) | 1.22 (1.16, 1.28) | <0.001 | 0.573 | 1.34 (1.27, 1.41) | <0.001 | 0.691 |
| Yes | 268 (1.05%) | 1.26 (1.16, 1.37) | <0.001 |  | 1.29 (1.17, 1.43) | <0.001 |  |
| BMI |  |  |  |  |  |  |  |
| <30 kg/m2 | 1,021 (0.33%) | 1.22 (1.16, 1.30) | <0.001 | 0.367 | 1.37 (1.29, 1.46) | <0.001 | 0.044 |
| ≥30 kg/m2 | 655 (0.65%) | 1.27 (1.19, 1.35) | <0.001 |  | 1.23 (1.14, 1.33) | <0.001 |  |

| **Aortic valve regurgitation** | | | | | | | |
| --- | --- | --- | --- | --- | --- | --- | --- |
| **Subgroup Analysis** |  | **PhenoAge acceleration** | | | **KDM-BA acceleration** | | |
|  | **Events, n(%)** | **HR (95% CI)** | **P-value** | **P-interaction** | **HR (95% CI)** | **P-value** | **P-interaction** |
| Chronological age (years) |  |  |  |  |  |  |  |
| ＜65 | 1,064 (0.32%) | 1.21 (1.15, 1.27) | <0.001 | 0.019 | 1.17 (1.10, 1.24) | <0.001 | <0.001 |
| ≥65 | 575 (0.74%) | 1.13 (1.05, 1.22) | 0.002 |  | 0.93 (0.87, 1.01) | 0.082 |  |
| Sex |  |  |  |  |  |  |  |
| Women | 692 (0.31%) | 1.21 (1.14, 1.29) | <0.001 | 0.242 | 1.05 (0.97, 1.15) | 0.22 | 0.846 |
| Men | 947 (0.50%) | 1.16 (1.10, 1.23) | <0.001 |  | 1.08 (1.02, 1.14) | 0.01 |  |
| College educated |  |  |  |  |  |  |  |
| No | 1,194 (0.43%) | 1.17 (1.11, 1.23) | <0.001 | 0.242 | 1.06 (0.998, 1.12) | 0.043 | 0.674 |
| Yes | 445 (0.34%) | 1.20 (1.11, 1.31) | <0.001 |  | 1.11 (1.02, 1.22) | 0.023 |  |
| Smoking status, n (%) |  |  |  |  |  |  |  |
| Never | 840 (0.38%) | 1.18 (1.11, 1.25) | <0.001 | 0.863 | 1.15 (1.07, 1.23) | <0.001 | 0.019 |
| Previous or Current | 799 (0.43%) | 1.17 (1.10, 1.24) | <0.001 |  | 1.01 (0.95, 1.08) | 0.657 |  |
| Alcohol intake frequency, n (%) | |  |  |  |  |  |  |
| Less than daily | 1,286 (0.40%) | 1.17 (1.12, 1.23) | <0.001 | 0.873 | 1.08 (1.02, 1.14) | 0.007 | 0.508 |
| Daily | 353 (0.42%) | 1.18 (1.07, 1.30) | 0.001 |  | 1.06 (0.95, 1.17) | 0.304 |  |
| Healthy physical activity |  |  |  |  |  |  |  |
| No | 773 (0.41%) | 1.20 (1.13, 1.27) | <0.001 | 0.271 | 1.09 (1.02, 1.17) | 0.011 | 0.434 |
| Yes | 866 (0.39%) | 1.15 (1.08, 1.23) | <0.001 |  | 1.05 (0.98, 1.13) | 0.129 |  |
| Hypertension |  |  |  |  |  |  |  |
| No | 728 (0.27%) | 1.12 (1.04, 1.21) | 0.004 | 0.814 | 1.06 (0.98, 1.15) | 0.122 | 0.740 |
| Yes | 911 (0.65%) | 1.15 (1.09, 1.22) | <0.001 |  | 1.02 (0.96, 1.09) | 0.465 |  |
| Dyslipidemia |  |  |  |  |  |  |  |
| No | 838 (0.29%) | 1.12 (1.04, 1.21) | 0.002 | 0.543 | 1.08 (1.01, 1.16) | 0.026 | 0.577 |
| Yes | 801 (0.68%) | 1.17 (1.11, 1.24) | <0.001 |  | 1.04 (0.97, 1.10) | 0.268 |  |
| Diabetes |  |  |  |  |  |  |  |
| No | 1,488 (0.39%) | 1.18 (1.13 ,1.24) | <0.001 | 0.294 | 1.07 (1.01, 1.12) | 0.014 | 0.803 |
| Yes | 151 (0.59%) | 1.12 (1 ,1.25) | 0.051 |  | 1.08 (0.94, 1.23) | 0.275 |  |
| BMI |  |  |  |  |  |  |  |
| <30 kg/m2 | 1,129 (0.37%) | 1.16 (1.09, 1.22) | <0.001 | 0.960 | 1.07 (1.01, 1.14) | 0.019 | 0.579 |
| ≥30 kg/m2 | 503 (0.50%) | 1.17 (1.09, 1.26) | <0.001 |  | 1.03 (0.95, 1.13) | 0.421 |  |

| **Mitral valve regurgitation** | | | | | | | |
| --- | --- | --- | --- | --- | --- | --- | --- |
| **Subgroup Analysis** |  | **PhenoAge acceleration** | | | **KDM-BA acceleration** | | |
|  | **Events, n(%)** | **HR (95% CI)** | **P-value** | **P-interaction** | **HR (95% CI)** | **P-value** | **P-interaction** |
| Chronological age (years) |  |  |  |  |  |  |  |
| ＜65 | 2,984 (0.90%) | 1.29 (1.26, 1.33) | <0.001 | 0.014 | 1.20 (1.16, 1.24) | <0.001 | 0.044 |
| ≥65 | 1,919 (2.46%) | 1.25 (1.21, 1.30) | <0.001 |  | 1.11 (1.06, 1.16) | <0.001 |  |
| Sex |  |  |  |  |  |  |  |
| Women | 2,163 (0.98%) | 1.27 (1.22, 1.31) | <0.001 | 0.620 | 1.15 (1.10, 1.21) | <0.001 | 0.951 |
| Men | 2,740 (1.45%) | 1.29 (1.26, 1.33) | <0.001 |  | 1.17 (1.13, 1.21) | <0.001 |  |
| College educated |  |  |  |  |  |  |  |
| No | 3,573 (1.29%) | 1.29 (1.26, 1.33) | <0.001 | 0.048 | 1.17 (1.13, 1.2) | <0.001 | 0.300 |
| Yes | 1,330 (1.00%) | 1.22 (1.16, 1.28) | <0.001 |  | 1.14 (1.08, 1.21) | <0.001 |  |
| Smoking status, n (%) |  |  |  |  |  |  |  |
| Never | 2,325 (1.04%) | 1.26 (1.22, 1.30) | <0.001 | 0.366 | 1.19 (1.15, 1.25) | <0.001 | 0.298 |
| Previous or Current | 2,578 (1.39%) | 1.3 (1.26, 1.33) | <0.001 |  | 1.14 (1.10, 1.18) | <0.001 |  |
| Alcohol intake frequency, n (%) | |  |  |  |  |  |  |
| Less than daily | 3,811 (1.17%) | 1.29 (1.26, 1.32) | <0.001 | 0.072 | 1.18 (1.15, 1.22) | <0.001 | 0.005 |
| Daily | 1,092 (1.31%) | 1.22 (1.16, 1.29) | <0.001 |  | 1.08 (1.02, 1.15) | 0.008 |  |
| Healthy physical activity |  |  |  |  |  |  |  |
| No | 2,349 (1.25%) | 1.29 (1.26, 1.33) | <0.001 | 0.186 | 1.15 (1.11, 1.20) | <0.001 | 0.870 |
| Yes | 2,554 (1.15%) | 1.26 (1.22, 1.30) | <0.001 |  | 1.17 (1.12, 1.22) | <0.001 |  |
| Hypertension |  |  |  |  |  |  |  |
| No | 2,168 (0.81%) | 1.17 (1.12, 1.22) | <0.001 | 0.001 | 1.10 (1.05, 1.15) | <0.001 | 0.098 |
| Yes | 2,735 (1.96%) | 1.28 (1.25, 1.31) | <0.001 |  | 1.14 (1.10, 1.18) | <0.001 |  |
| Dyslipidemia |  |  |  |  |  |  |  |
| No | 2,461 (0.85%) | 1.18 (1.14, 1.23) | <0.001 | <0.001 | 1.13 (1.08, 1.18) | <0.001 | 0.204 |
| Yes | 2,442 (2.07%) | 1.29 (1.26, 1.32) | <0.001 |  | 1.15 (1.11, 1.20) | <0.001 |  |
| Diabetes |  |  |  |  |  |  |  |
| No | 4,340 (1.13%) | 1.25 (1.21, 1.28) | <0.001 | 0.032 | 1.12 (1.09, 1.16) | <0.001 | <0.001 |
| Yes | 563 (2.20%) | 1.31 (1.25, 1.37) | <0.001 |  | 1.28 (1.19, 1.37) | <0.001 |  |
| BMI |  |  |  |  |  |  |  |
| <30 kg/m2 | 3,388 (1.10%) | 1.25 (1.21, 1.28) | <0.001 | 0.085 | 1.14 (1.10, 1.18) | <0.001 | 0.477 |
| ≥30 kg/m2 | 1,495 (1.49%) | 1.3 (1.26, 1.35) | <0.001 |  | 1.16 (1.10, 1.22) | <0.001 |  |

Model 1 adjusted for sex, ethnicity, education, smoking status, alcohol intake frequency, physical activity, and the Townsend Deprivation Index.

## Table S6. Multicollinearity diagnostics of risk factors in Primary Cohort.

|  | **Aortic valve stenosis** | | **Aortic valve regurgitation** | | **Mitral valve regurgitation** | |
| --- | --- | --- | --- | --- | --- | --- |
|  | **VIF (Tolerance)** | **VIF (Tolerance)** | **VIF (Tolerance)** | **VIF (Tolerance)** | **VIF (Tolerance)** | **VIF (Tolerance)** |
| PhenoAge acceleration | 1.26 (0.79) | - | 1.21 (0.83) | - | 1.26 (0.79) | - |
| KDM-BA acceleration | - | 1.09 (0.92) | - | 1.08 (0.93) | - | 1.09 (0.92) |
| Age | 1.12 (0.90) | 1.12 (0.90) | 1.16 (0.86) | 1.16 (0.86) | 1.17 (0.86) | 1.17 (0.86) |
| Sex | 1.10 (0.91) | 1.12 (0.89) | 1.09 (0.92) | 1.10 (0.91) | 1.10 (0.91) | 1.12 (0.89) |
| White Ethnicity | 1.03 (0.97) | 1.03 (0.97) | 1.05 (0.95) | 1.05 (0.95) | 1.04 (0.96) | 1.04 (0.96) |
| College education | 1.06 (0.95) | 1.05 (0.95) | 1.06 (0.94) | 1.06 (0.94) | 1.06 (0.94) | 1.06 (0.94) |
| Smoking status | 1.11 (0.90) | 1.12 (0.90) | 1.10 (0.91) | 1.11 (0.90) | 1.11 (0.90) | 1.12 (0.90) |
| Alcohol frequency | 1.10 (0.91) | 1.10 (0.91) | 1.10 (0.91) | 1.10 (0.91) | 1.10 (0.91) | 1.10 (0.91) |
| Healthy physical activity | 1.02 (0.98) | 1.02 (0.98) | 1.01 (0.99) | 1.01 (0.99) | 1.01 (0.99) | 1.02 (0.98) |
| Townsend deprivation score | 1.08 (0.93) | 1.08 (0.92) | 1.08 (0.92) | 1.09 (0.92) | 1.08 (0.93) | 1.08 (0.92) |
| Hypertension | 1.79 (0.56) | 1.83 (0.55) | 1.77 (0.56) | 1.81 (0.55) | 1.83 (0.55) | 1.87 (0.53) |
| Obesity | 1.03 (0.97) | 1.04 (0.96) | 1.02 (0.98) | 1.02 (0.98) | 1.03 (0.97) | 1.03 (0.97) |
| Dyslipidemia | 2.00 (0.50) | 2.04 (0.49) | 1.99 (0.50) | 2.03 (0.49) | 2.07 (0.48) | 2.13 (0.47) |
| Diabetes | 2.51 (0.40) | 2.71 (0.37) | 2.31 (0.43) | 2.43 (0.41) | 2.58 (0.39) | 2.75 (0.36) |
| Osteoporosis | 1.03 (0.98) | 1.03 (0.98) | 1.02 (0.98) | 1.02 (0.98) | 1.03 (0.98) | 1.02 (0.98) |
| Coronary artery disease | 1.36 (0.73) | 1.39 (0.72) | 1.37 (0.73) | 1.39 (0.72) | 1.45 (0.69) | 1.49 (0.67) |
| Heart failure | 1.08 (0.92) | 1.09 (0.91) | 1.06 (0.95) | 1.06 (0.94) | 1.18 (0.85) | 1.21 (0.83) |
| Atrial fibrillation | 1.09 (0.91) | 1.10 (0.91) | 1.12 (0.89) | 1.13 (0.89) | 1.19 (0.84) | 1.21 (0.83) |
| Cardiomyopathy | 1.02 (0.98) | 1.03 (0.97) | 1.03 (0.97) | 1.03 (0.97) | 1.05 (0.95) | 1.06 (0.94) |
| Chronic kidney disease | 1.02 (0.98) | 1.03 (0.97) | 1.02 (0.98) | 1.02 (0.98) | 1.02 (0.98) | 1.04 (0.97) |
| Antidiabetic medication | 2.46 (0.41) | 2.63 (0.38) | 2.27 (0.44) | 2.38 (0.42) | 2.54 (0.39) | 2.69 (0.37) |
| Antithrombotic medication | 1.59 (0.63) | 1.65 (0.61) | 1.61 (0.62) | 1.66 (0.60) | 1.68 (0.59) | 1.75 (0.57) |

VIF, Variance Inflation Factor.

## Figure S1. Adjusted survival curves and dose–response relationships between biological age accelerations and the risk of aortic valve regurgitation-related and mitral valve regurgitation-related events.


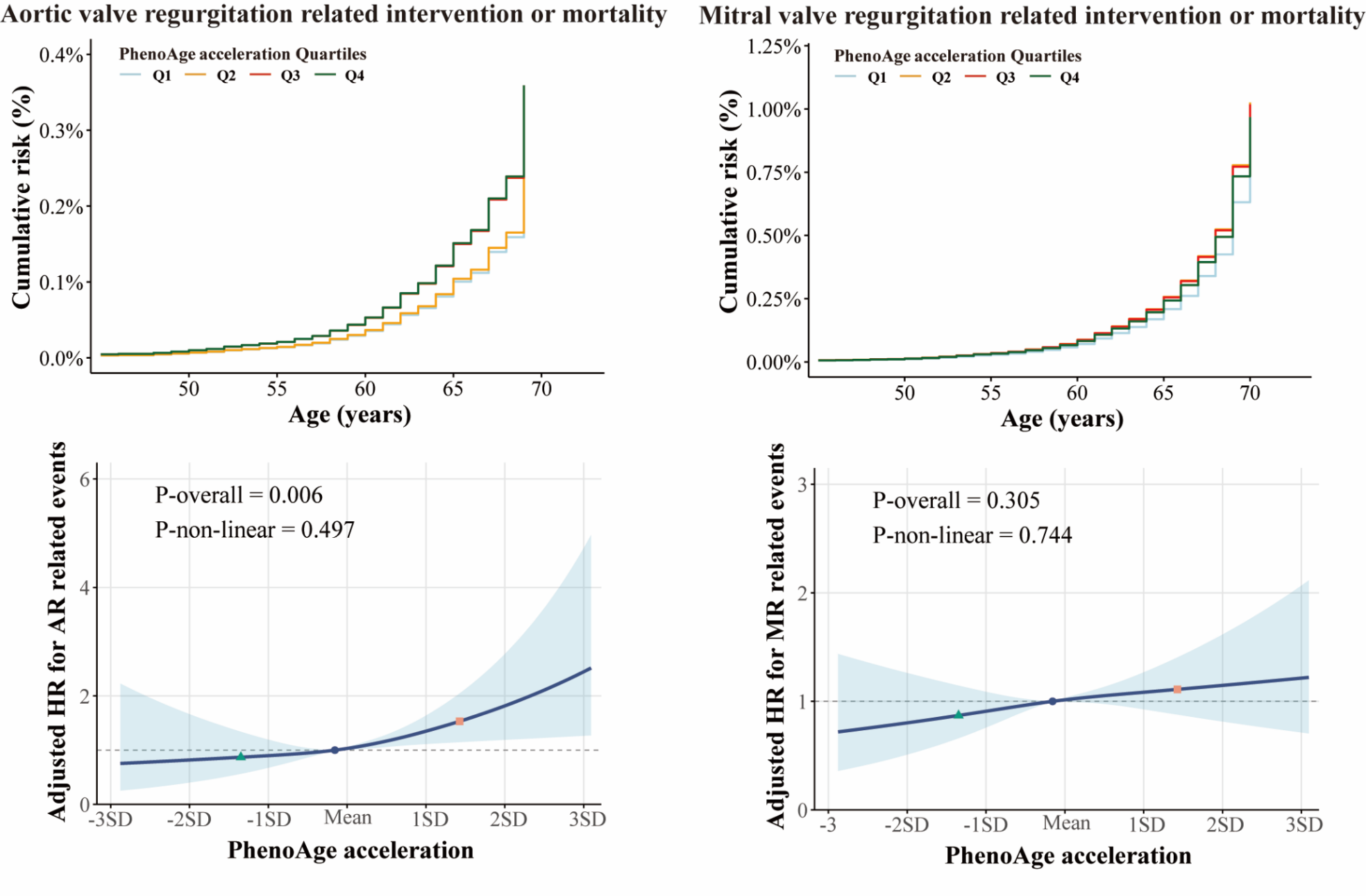


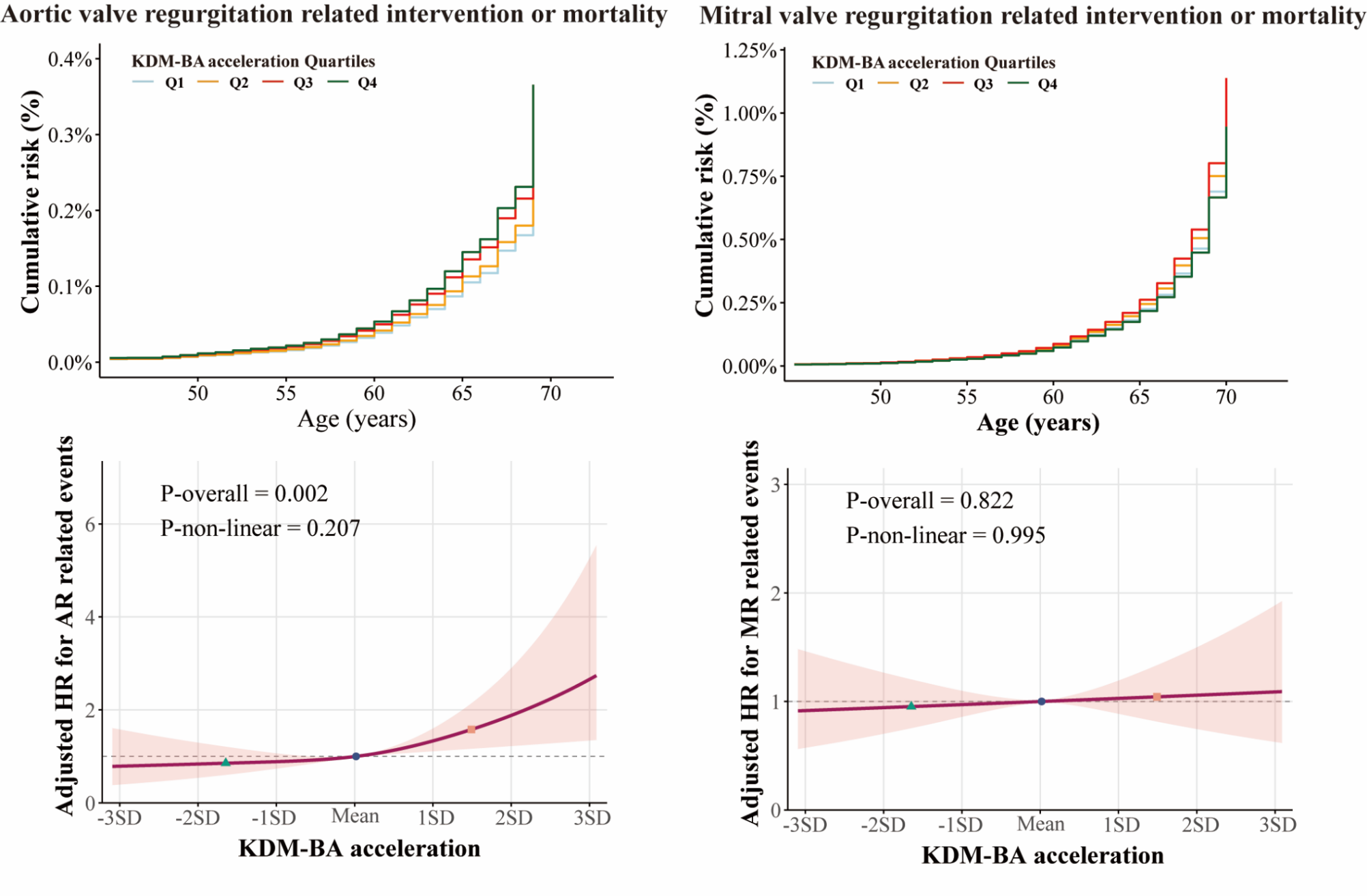

Models were adjusted for age, sex, ethnicity, education, smoking status, alcohol intake frequency, physical activity, and Townsend deprivation index. Upper panel: The adjusted cumulative risk plot was generated using age as the timescale. Lower panel: The restricted cubic spline plot was generated using follow-up duration as the timescale. Solid line: Point estimate; Shaded area: 95% confidence interval; Dots: 5th, 50th, and 95th percentiles.

##
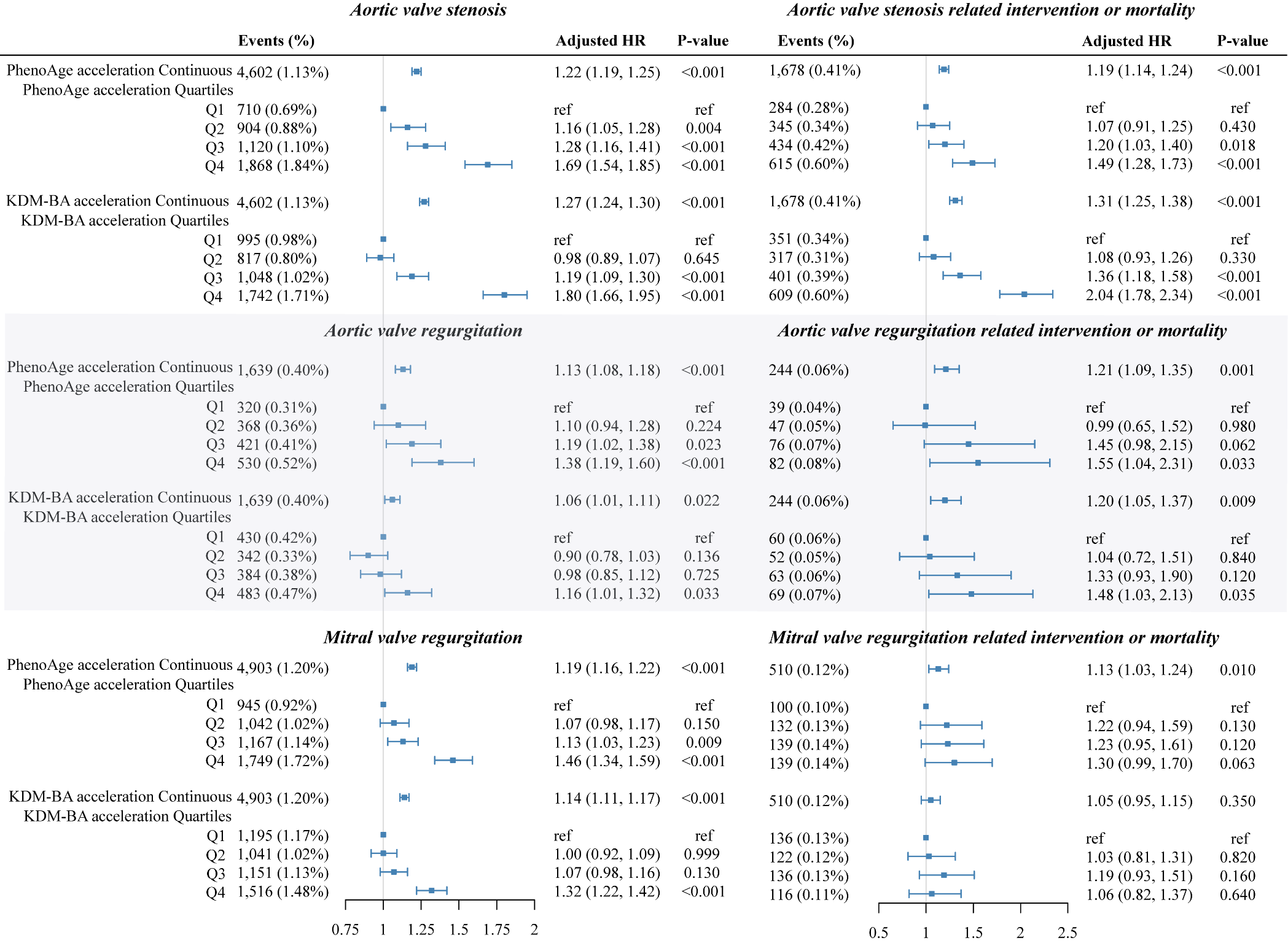
Figure S2. Adjusted hazard ratio for degenerative valvular heart disease across BAAs quartiles (Primary Cohort; Model 2).

The adjusted hazard ratio for valvular heart disease (VHD) was estimated using the Cox proportional hazards model, while the adjusted hazard ratio for VHD-related events was derived using the Fine and Gray competing risks regression model, which accounts for death from other causes as a competing event.

Model 2 adjusted for age, sex, ethnicity, education, smoking status, alcohol intake frequency, healthy physical activity, Townsend deprivation index, clinical comorbidities (hypertension, obesity, dyslipidemia, diabetes, osteoporosis, coronary artery disease, heart failure, atrial fibrillation, cardiomyopathy, and chronic kidney disease), and medications (anti-diabetic, and antithrombotic medication).

##
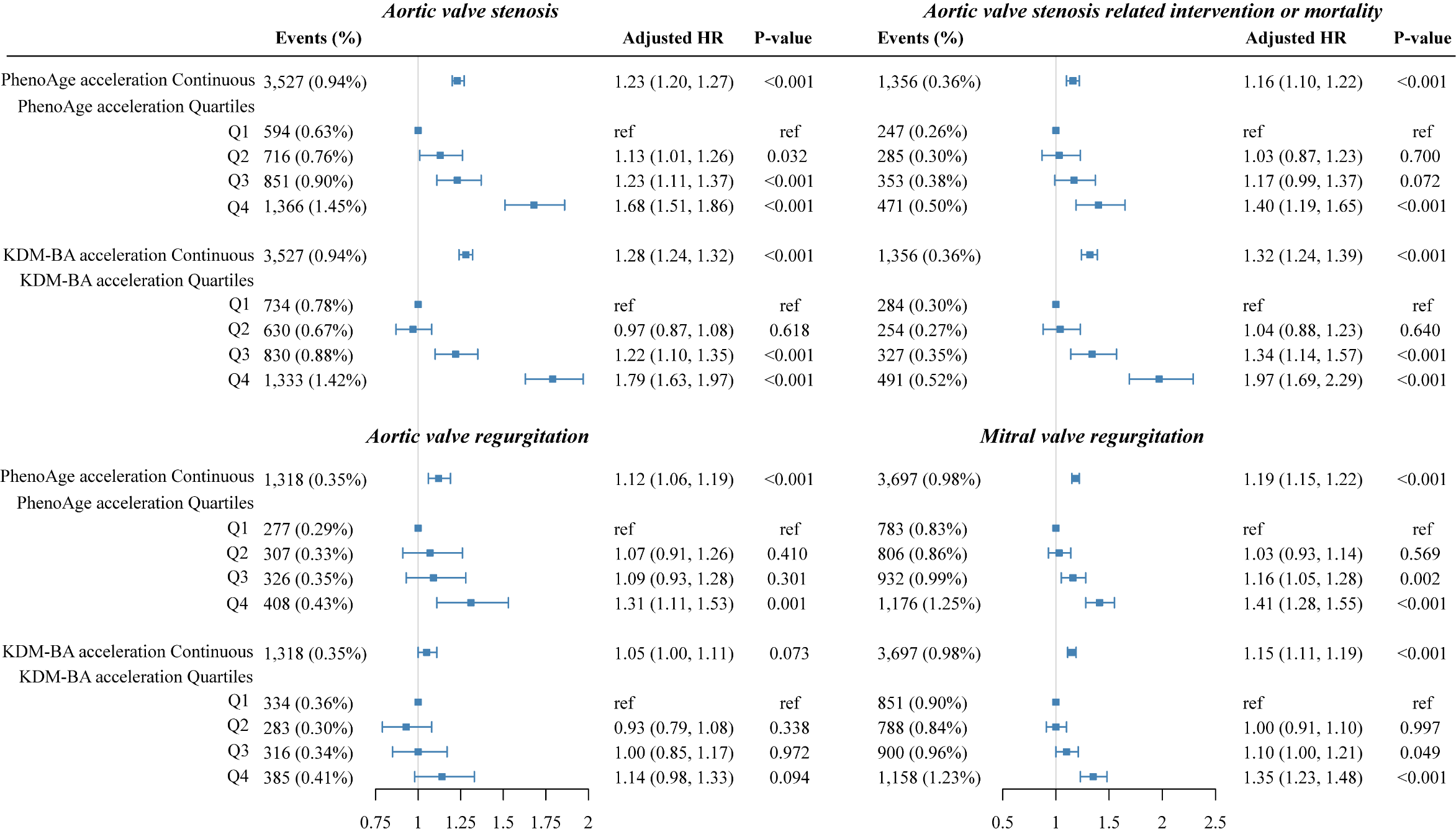
Figure S3. Adjusted hazard ratio for degenerative valvular heart disease across BAAs quartiles excluding participants with baseline cardiovascular comorbidities (coronary artery disease, heart failure, atrial fibrillation, cardiomyopathy, and chronic kidney disease) (Analytic Cohort 2; Model 2).

The adjusted hazard ratios for aortic stenosis (AS), aortic valve regurgitation and mitral valve regurgitation were estimated using the Cox proportional hazards model. The adjusted hazard ratio for AS-related events was derived using the Fine and Gray competing risks regression model, which accounts for death from other causes as a competing event.
Model 2 adjusted for age, sex, ethnicity, education, smoking status, alcohol intake frequency, healthy physical activity, Townsend deprivation index, clinical comorbidities (hypertension, obesity, dyslipidemia, diabetes, and osteoporosis), and medications (anti-diabetic, and antithrombotic medication).

##
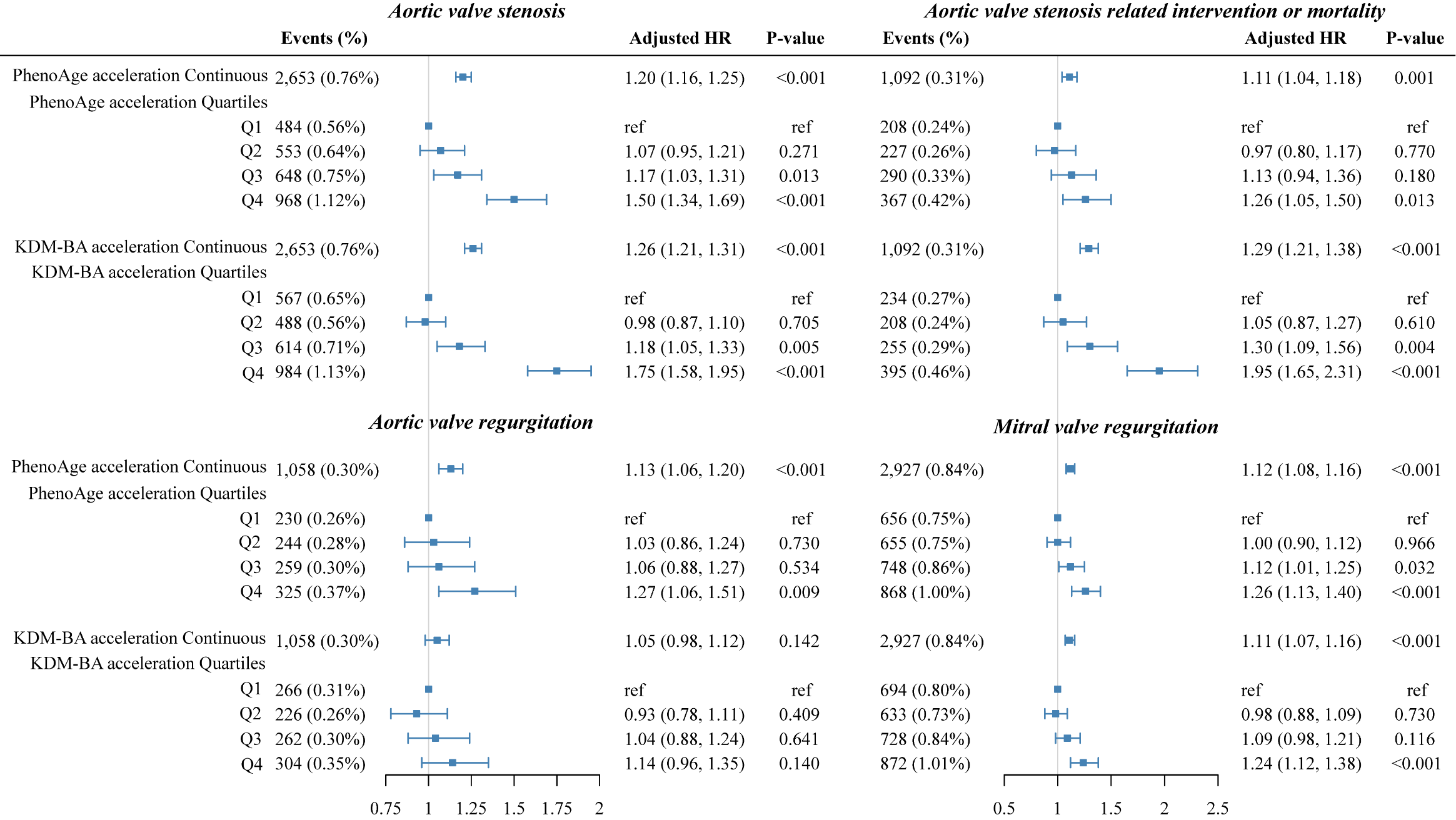
Figure S4. Adjusted hazard ratio for degenerative valvular heart disease across BAAs quartiles excluding participants with baseline cardiovascular comorbidities and with follow-up duration <2 years. (Analytic Cohort 3; Model 2)

Baseline cardiovascular comorbidities included coronary artery disease, heart failure, atrial fibrillation, cardiomyopathy, and chronic kidney disease. The adjusted hazard ratios for aortic stenosis (AS), aortic valve regurgitation and mitral valve regurgitation were estimated using the Cox proportional hazards model. The adjusted hazard ratio for AS-related events was derived using the Fine and Gray competing risks regression model, which accounts for death from other causes as a competing event.
Model 2 adjusted for age, sex, ethnicity, education, smoking status, alcohol intake frequency, healthy physical activity, Townsend deprivation index, clinical comorbidities (hypertension, obesity, dyslipidemia, diabetes, and osteoporosis), and medications (anti-diabetic, and antithrombotic medication).
